# Care for Joy: Evaluation of a Humor Intervention and Its Effects on Stress, Flow Experience, Work Enjoyment, and Meaningfulness of Work

**DOI:** 10.3389/fpubh.2021.667821

**Published:** 2021-07-02

**Authors:** Marek Bartzik, Andreas Bentrup, Susanne Hill, Maria Bley, Eckart von Hirschhausen, Gerrit Krause, Peter Ahaus, Angelika Dahl-Dichmann, Corinna Peifer

**Affiliations:** ^1^Research Group Work and Health, Department of Psychology, University of Lübeck, Lübeck, Germany; ^2^Stiftung Humor Hilft Heilen (Foundation Humour Helps Healing), Bonn, Germany; ^3^Alexianer GmbH, Münster, Germany; ^4^Alexianer GmbH, Berlin, Germany

**Keywords:** humor, intervention, stress, flow experience, work enjoyment, meaningfulness of work, nurse

## Abstract

The media increasingly speak of a care crisis. Systematic support is needed to prepare nursing apprentices for the high demands of their profession and to reduce the number of nurses who finally quit. Particularly in stressful jobs like nursing, humor as a coping strategy can have a beneficial effect on perceived stress and overall work enjoyment. In this study, we used a humor intervention among nursing staff in training and evaluated its effects on humor, stress, work enjoyment, the meaningfulness of work, and flow experience. The sample consists of 104 nurses in training. The intervention group received a 3-h humor intervention, while the control group received no intervention. Positive and negative affect were measured immediately before and after the intervention. Humor was measured before the intervention (t_0_) and again 6 months later (t_1_); at t_1_, we again measured humor and also stress, work meaningfulness, work enjoyment, and flow experience. Our analyses showed a beneficial change in positive and negative affect right after the intervention. By means of repeated measures ANOVA we could further confirm an effect of the intervention on reported humor 6 months later. Humor mediated positive effects of the humor intervention on perceived meaningfulness of work, work enjoyment, and on the frequency of flow at work. Also, we found a significant negative relationship between humor and stress measured at t_1_. The results of this study confirm the effectiveness of humor interventions in promoting humor, and, through this, the meaningfulness of work, work enjoyment, and the frequency of flow experience. Implications of the use of humor interventions in the nursing profession are discussed.

## Introduction

Media often speak of a care crisis. Due to demographic change and medical progress, a considerable shortage of skilled workers in the nursing profession is predicted for the future (1–3). Reasons for this are the increasing age of the patients and the increasing age of the nurses themselves; also, it is expected that fewer young nurses will enter the profession in the future (4). We further know that nursing staff are under great physical and psychological strain in their profession, and there has been an increase in absenteeism and the intention to terminate (5). Accordingly, there is a need for action regarding the working conditions of nursing staff to make the profession more attractive for young nurses and to reduce fluctuations. In particular, the increased number of terminations by nurses can have extensive consequences, such as high economic costs, reduced well-being of the remaining nurses or lower satisfaction with care from the patient's perspective (6). Experienced stress at work can be a reason for termination intentions (7, 8), and also for burnout among nurses (9). Not only does burnout negatively impact health among nurses (10), but patients also show higher satisfaction with care when nurses report lower burnout levels (11). Accordingly, there is a need for interventions that help nurses to cope with their work-related stress (12, 13). Research has identified the use of humor as a promising strategy to deal with stress (14–18). The aim of our study is, thus, to evaluate the effectiveness of a humor intervention for nurses in training. More specifically, we look at the effects of the intervention on sense of humor, and, in consequence, on work experience, including perceived stress, work enjoyment, frequency of flow experience, and perceived meaningfulness of work as mediated by one's sense of humor. The humor intervention was conducted with nurses in training and their results were compared to a control group without intervention.

### Humor

The construct of humor has been described in the field of Positive Psychology (19) and is a very complex, multidimensional phenomenon. There are various approaches to its definition and classification (20). One such approach was that of Martin (21), according to whom humor is a process with cognitive, emotional, and interpersonal aspects (21); it can be defined as

“… a broad term that refers to anything that people say or do that is perceived as funny and tends to make others laugh, as well as the mental processes that go into both creating and perceiving such an amusing stimulus, and also the affective response involved in the enjoyment of it. From a psychological perspective, the humor process can be divided into four essential components: (1) a social context, (2) a cognitive-perceptual process, (3) an emotional response, and (4) the vocal-behavioral expression of laughter.” [(21), p. 5].

In our study we refer to the sense of humor: this refers to the habit of laughing at humor and using humor more often than the average person (22–24). Sense of humor is defined as:

“… a habitual behavior pattern (tendency to laugh frequently, to tell jokes and amuse others, to laugh at other people's jokes), an ability (ability to create humor, to amuse others, to “get the joke,” to remember jokes), a temperamental trait (habitual cheerfulness), an aesthetic response (enjoyment of particular types of humorous material), an attitude (positive attitude toward humor and humorous people), a world view (bemused outlook on life), or a coping strategy (tendency to maintain a humorous perspective in the face of adversity).” [(25), p. 315].

We find humor not only as an independent construct, but also in other concepts of Positive Psychology, such as character strengths (19, 26, 27). Character strengths are morally valued aspects of one's personality. Examples are creativity, wisdom, kindness, bravery, modesty, and many more, including humor (26). Character strengths are described as relatively stable, but they can also be changed. The definition of sense of humor shows similarities to the definition of humor as a character strength (26), and their positive relationship was confirmed in an empirical study that found correlations between the two (28).

Sense of humor is divided into six different sense of humor habits, which can be described as enjoyment of humor, laughter, verbal humor, finding humor in everyday life, laughing at yourself, and humor under stress (17, 29). The six sense of humor habits together represent a total value of the sense of humor, but this should not only be seen as a one-factor model; rather, the six different sense of humor habits each provide unique information and should, thus, also be individually reported (30).

Humor has many functions within and between persons in the work context (20). For example, humor has an important function in communication (31) and it can increase well-being (32–35) and positive affect (32, 36, 37). Also, humor has relationships between r = 0.23 and r = 0.43 with each element of the PERMA Model (38). The PERMA Model (39) describes five pillars of well-being, which are: “positive emotions,” “engagement,” “positive relationships,” “meaning,” and “accomplishment” (39). Building upon the positive relationship between humor, positive emotions and well-being, including the five pillars of the PERMA model, research has found that humor can function as a coping strategy in dealing with stress (25, 40, 41). Increased well-being is associated with greater resilience and, thus, can acts as a protective factor against stress (42). Also, the positive emotions elicited by humor in the moment are not compatible with stress, which supports a re-framing of the situation and successful coping (40).

### Humor in the Care Context

Humor as a form of communication is a helpful tool for patient-centered care (43). For example, humor can be used to build and maintain a relationship (44). Literature suggests that humor improves the understanding of therapeutic concepts and leads to a higher acceptance and therapy adherence; this further results in reduced challenges for the care givers (45). Use of humor by nurses is interpreted by patients as a positive characteristic of a nurse and is also an important aspect of patient/nurse interaction (46). Humor in the nursing context improves communication and also increases trust between nurse and patient (47, 48). Humor can also create a sense of cohesion not only between patients and nurses, but also among colleagues. Further, humor helps one to deal with difficult situations and difficult patients (49). A literature review looked at the positive aspects of humor in healthcare and concluded that nurses should be aware of their own humor and use it to interact with patients (50). In general, patients' anxiety can be reduced through the use of humor (47, 51); at the same time, patients feel supported by humorous nursing interventions with regard to their health and the healing process (47). The use of humor in the nursing profession is a complex nursing intervention that requires a lot of creative energy and also cognitive skills in the interaction between patients and nurses (47, 48). It is recommended that the use of humor as a nursing intervention should be tailored to the individual patient (48); also, the right timing of the use of humor is important (52).

Due to the complexity of nursing interventions, special training for the use of humor in the nursing context should be conducted (48). For this reason, the humor training program “*Care for Joy*” for nurses in training has been developed. It is designed to prepare nursing staff in training for the reported high stress of their profession. This study deals with the first module of the “*Care for Joy*” training. We aim to examine the effects of this first module on sense of humor and the corresponding six sense of humor habits (17) in an intervention group as compared to a control group.

### Humor Training

To use the full potential of humor in the health care context, humor trainings are promising interventions. Importantly, sense of humor is not stable over time and is considered changeable (41). Studies show that sense of humor can be trained and developed (53–55). An already known training program “*The 7 Humor Habits Program*” was developed by McGhee (17) and provides the basis for a further practical training for psychiatric-psychotherapeutic practice, which is also suitable for healthy individuals (53). “*The 7 Humor Habits Program*” aims to build and strengthen humor in everyday life as a skill for successful stress management (17). The effectiveness of humor training like “*The 7 Humor Habits Program*” has already been confirmed in studies. For example, it has been shown that humor training increases sense of humor, self-efficacy, positive thinking, optimism, and happiness, and decreases negative thinking, depression, anxiety, and stress (33, 54–56).

### Humor Training in the Care Context

While humor trainings have been successfully tested in the field, there is still a lack of profession-specific humor trainings in the care context. For example, “*The 7 Humor Habits Program*” is not designed for a specific group of participants, but for all those who have forgotten to use humor in everyday life and have lost their playful attitude in life (17). Due to the complexity of humor in the care context, caregivers should receive systematic support in the form of training (48), which takes into account job-specific situations, such as contact with patients in difficult circumstances. In order to develop the sense of humor for nurses in training, we have therefore created a humor training for this specific target group. The training and the individual humor interventions have been developed with a problem-based approach. Problem-based training has its origin in medical school and is characterized by the fact that learning is an active process with direct reference to problems in practice (57). It has been shown that learning is facilitated by using problem-based methods (57); at the same time, problem-based training shows the learners how they can apply what they have learned in practice (58)—a key factor for successful transfer after training. Also a meta-analysis shows that problem-based training has positive effects on the acquisition of skills, i.e., the application of knowledge (59). Based on this, and based on the above mentioned studies that show that the sense of humor can be trained (33, 54, 55), we assume that our humor intervention increases the sense of humor and the six sense of humor habits.

*Hypothesis 1: The humor intervention has a positive effect on the nurses' sense of humor and on the six sense of humor habits*.

### Perceived Stress

A well-known stress model is the *transactional model of stress and coping* (60), which is based on a primary assessment of a stressor and classifies this stressor as positive, negative or irrelevant. A negatively assessed stressor is subjected to a secondary assessment, in which resources are compared with the demands of the stressor. Lack of resources can lead to stress (60). In the *transactional model of stress and coping* (60), humor can act as a coping strategy through cognitive appraisal and subsequent behavior (61). Stress-based emotions and stressful person-environment relations can be regulated by humor. This was shown in a qualitative study in which nurses in training used humor as a coping strategy to cope with stressful person-environment relations (e.g., dealing with patients who violate social norms) and to achieve positive affect as an outcome (61). Even in extraordinary times such as the COVID-19 pandemic, humor has been shown to be an effective coping strategy (62) and this was also found for nurses (63, 64). People with a greater sense of humor can manage stress more effectively (16). A review article on humor in medicine concludes that humor can reduce stress in medical professionals and patients (14). Humor creates positive emotions that are incompatible with stress and that thus facilitate coping (40). Especially in stressful occupations such as nursing, humor as a coping strategy can have a positive effect on the perceived stress level (14–17, 61). Therefore, increases in the sense of humor due to our humor intervention should translate into reduced levels of perceived stress.

*Hypothesis 2: An increased sense of humor mediates negative effects of the humor intervention on perceived stress*.

### Work Enjoyment During Practical Training

The training of nurses alternates between phases of theoretical and practical training. Work enjoyment during practical training can be defined as “… the degree to which individuals work because they find the work itself intrinsically interesting or pleasurable” [Johnstone and Johnston, 2005; McMillan et al., 2002; Spence and Robbins, 1992 as cited in (65), p. 1656]. One important reason why nurses enjoy their work is because they enjoy interacting with and caring for patients, which is at the same time one reason why they stay in the nursing profession (66). Studies show that humor has an impact on positive affect (32, 36, 37, 67). There are strong links between the concepts of work enjoyment, positive affect and job satisfaction (68–70), and work enjoyment has even been used as a dimension in the assessment of job satisfaction [MOAQ (71)]. A meta-analysis shows that humor is associated with job satisfaction (72) and further that day-related job satisfaction can predict humor production the following day (73). By implementing humor in the work context, work enjoyment should thus increase (74). This relationship has not yet been shown, however, in the context of health care workers. Therefore, we aim to test if the increase of sense of humor caused by the humor intervention will lead to increased work enjoyment.

*Hypothesis 3: Sense of humor mediates a positive effect of the humor intervention on work enjoyment*.

### Flow Experience

Flow is described as a pleasant and rewarding state of full absorption during the performance of activities, and it is facilitated clear feedback, clear goals and a balance of demands and abilities (75). Flow can also be assigned to the *PERMA Model* (39), under the pillar of engagement (39, 76).

Flow promotes well-being (77–81) and performance (79, 82–84). Like humor (61), the *transactional model of stress and coping* (60) can also be associated with flow (85). In interview studies, the constructs of fun at work and flow experience were implemented into a theoretical framework; in those studies, fun at work was described as flow-promoting (86, 87). In a quantitative study, a correlation between flow and humor could also be shown (88). In line with this, self-reported humor and the element “*engagement*” from the *PERMA Model* have been shown to correlate (38). To the best of our knowledge, there are no other studies that have investigated the direct relationship between sense of humor and flow.

Studies show that flow is positively associated with positive affect (89–91) and negatively associated with negative affect (89). Fun is described as a factor that can promote flow in everyday work (87, 92). Also, it was found that having previous positive affect was a significant predictor of increased flow (93). As humor promotes positive affect (32, 36, 37, 67), promoting the sense of humor should positively affect flow-experience (87, 89, 91–93). Based on this assumption, we propose in hypothesis 4 that sense of humor can be increased by our humor intervention and that sense of humor acts as a mediator to increase the frequency of flow experience at work.

*Hypothesis 4: Sense of humor mediates a positive effect of the humor intervention on flow frequency*.

### Perceived Meaningfulness of Work

In the *Job-Characteristics Model* (94) the perceived meaningfulness of work is defined as “The degree to which the individual experiences the job as one which is generally meaningful, valuable and worthwhile” [(94), p. 256]. Whether or not work is considered meaningful is the result of an individual's subjective assessment (95). Various factors affect the perceived meaningfulness of work, which are the self, others, the work and its context, and spiritual life (95). The term “meaning” is associated with the identity of individuals and thus also with one's own work (96). Accordingly, we understand meaningful work in the nursing profession as a subjective assessment of the general meaningfulness of the work, the importance of the work for one's own identity, and the significance of the work for others and for society as a whole.

Employees who consider their work to be meaningful feel better at work, report fewer signs of depressive moods, feel needed at work and at the same time feel part of a group (97). The perceived importance of work has a positive effect on well-being (97–99). In a study with nurses, it was found that the nursing profession is perceived as meaningful and that this perception helps to deal with difficult challenges in the work environment. Also, nurses who evaluate their work as meaningful are less dependent on positive feedback regarding their work from patients or their relatives (100).

Humor as a component of character strengths is assigned to the category of transcendence strengths, which are defined as “strengths that (…) provide meaning” [(26), p. 30]. According to this definition, humor should also be able to provide meaning. To the best of our knowledge, there are no studies yet that investigate the effects of humor on the perceived meaningfulness of work. Based on the assumption that humor can generate meaning, we derive the hypothesis that our humor intervention enhances the perceived meaningfulness of work as mediated by an increased sense of humor.

*Hypothesis 5: Sense of humor mediates a positive effect of the humor intervention on the perceived meaningfulness of work*.

### Summary of our Hypotheses

In sum, we examine the long-term effects of the humor intervention on the sense of humor and its six sense of humor habits (*hypothesis 1*), and the resulting effects on perceived stress (*hypothesis 2*), work enjoyment (*hypothesis 3*), the frequency of flow experience (*hypothesis 4*), and perceived meaningfulness of work (*hypothesis 5*).

To test these hypotheses, we conducted a humor intervention with nurses in training (intervention group) while a comparable control group received no intervention. We measured sense of humor before and 6 months after the humor intervention to test our hypothesis of whether the humor intervention has long-term effects on sense of humor and the six sense of humor habits. We also examined if the increased sense of humor (as a mediator) translates into reduced stress, and increased work enjoyment, frequency of flow experience, and perceived meaningfulness of work.

### Further Evaluation of the Humor Intervention

In addition to testing the above described hypotheses, we evaluated the reactions of the participants to the humor intervention with regard to their attitudes toward the humor intervention, their subjective enjoyment during the humor intervention, the perceived usefulness for their work, and the perceived difficulty of the humor intervention. We also investigated the immediate effects of the humor intervention on our participants' positive and negative affect directly before compared to directly after the intervention. Furthermore, we examined if the acute change in positive affect and negative affect due to the humor intervention was related to the sense of humor and its subscales as well as on perceived stress, work enjoyment, frequency of flow experience, and perceived meaningfulness of work at t_1_.

## Methods

### Participants and Design

The participants were nurses in training who received a 3-h humor intervention [intervention group (IG)] or no intervention [control group (CG)]. In contrast to the IG, the CG did not receive any intervention. Data collection took place at two different nursing schools of the same health care provider, so that for both groups, the schools' curriculum is identical. The sample was composed of nurses in training of the same cohort and in the same year of training.

The participants completed questionnaires a few days before the training (t_0_) and 6 months later (t_1_) to evaluate longterm effects of our intervention. The control group completed the same questionnaires in the same period of time. The sample consists at t_0_ in total of *N* = 104 (85 females, 18 males, 1 not reported, *M*_age_ = 19.96, *SD*_age_ = 2.563), of which *N*_IGt1_ = 71 belonged to the intervention group (63 females, 7 males, 1 not reported, *M*_age_ = 19.77, *SD*_age_ = 1.578) and *N*_CGt0_ = 33 belonged to the control group (22 females, 11 males, *M*_age_ = 20.38, *SD*_age_ = 3.966). At t_1_, the sample consisted of *N*_*t*1_ = 94 (74 females, 20 males, *M*_age_ = 21.06, *SD*_age_ = 3.144), of which *N*_IGt1_ = 63 belonged to the intervention group (53 females, 10 males, *M*_age_ = 20.85, *SD*_age_= 2.708) and *N*_CGt1_ = 31 belonged to the control group (21 females, 10 males, *M*_age_ = 21.59, *SD*_age_ = 4.031). For an overview of the sample, see Table 1.

**Table 1:** Overview sample at t_0_ and t_1_ (*N* and mean and standard deviation of age).

**Measuring points**	**Overall sample**	**Control group (CG)**	**Intervention group (IG)**
Measuring point t_0_	*N* = 104, *M*_age_ = 19.96, *SD*_age_ = 2.563	*N*_(CG)_ = 33, *M*_age(CG)_ = 20.38, *SD*_age(CG)_ = 3.966	*N*_(IG)_ = 71, *M*_age(IG)_ = 19.77, *SD*_age(IG)_ = 1.578
Measuring point t_1_	*N* = 94, *M*_age_ = 21.06, *SD*_age_ = 3.144	*N*_(CG)_ = 31, *M*_age(CG)_ = 21.59, *SD*_age(CG)_ = 4.031	*N*_(IG)_ = 63, *M*_age(IG)_ = 20.85, *SD*_age(IG)_ = 2.708

*CG, control group; IG, intervention group; Measuring points: t_0_ = Baseline; t_1_ = 6 months after the humor intervention*.

### Procedure

**Figure 1:**
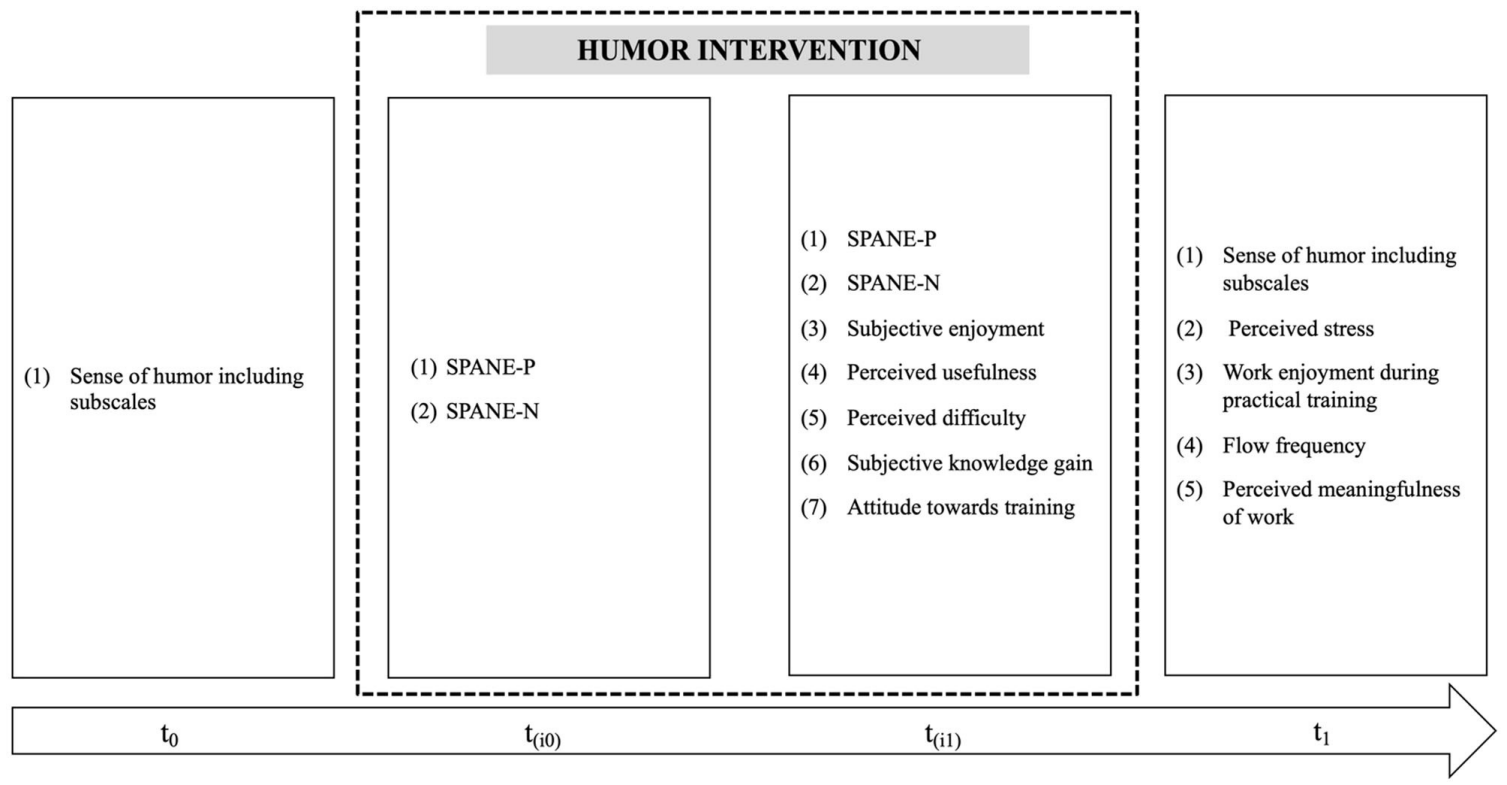
Study variables and measuring points. Measuring points: t_0_ = Baseline; t_1_ = 6 months after the humor intervention; t_(i0)_ = directly before the humor intervention; t_(i1)_ = directly after the humor intervention; SPANE-P, positive affect; SPANE-N, negative affect.

A few days before the humor intervention took place, all participants (IG + CG) completed a questionnaire that assessed their sense of humor baseline (t_0_) including the six sense of humor subscales. Six months after the intervention (t_1_) we measured again in both groups the sense of humor as well as the perceived stress, work enjoyment, frequency of flow experience, and meaningfulness of work. The intervention group additionally completed short questionnaires immediately before (t_i0_) and after the training (t_i1_) to assess changes in positive (*SPANE-P*) and negative affect (*SPANE-N*). For t_i1_ we additionally measured questions to evaluate the humor intervention with the “*Training Evaluation Inventory (TEI)*” (Level 1: reactions and Level 2: learning and attitude). An overview of the measurement points and the study variables can be seen in Figure 1. Our study was approved by the local ethics committee at the Ruhr University Bochum, Germany.

#### Humor Intervention

The humor intervention addresses humor and communication techniques to create a positive relationship with the patient, the patient's relatives as well as with colleagues. It combines practical exercises (e.g., emotion recognition) with subsequent theoretical input, and then reflects on how to translate the learnings into practice. The intervention aims at sensitizing the participants to recognize individual situations of patients in order to then adequately respond to them. The exercises are inspired by scientifically validated exercises on communication and emotion recognition and by positive psychological interventions (e.g., giving compliments) in combination with clown techniques and exercises from the field of theater. The humor intervention was conducted in a 3-h session in a classroom at the nursing school. The training that we used had been developed 6 years prior by the foundation “Humor Hilft Heilen” (Humor Helps Healing) and has been conducted with over 10,000 participants from health care. Also, this training has already been conducted in nursing schools for 3 years. The training is given by humor trainers of the foundation “Humor Hilft Heilen” (Humor Helps Healing). Further modules were developed for the “Care for Joy” project, which are carried out at 6-month intervals over a period of 3 years. As only the evaluation of the first module has been completed so far, the further developed modules are not the subject of this evaluation. The control group did not receive the humor intervention. In terms of content, the humor intervention defined humor and taught basic humorous communication skills in the context of the nursing profession. Also, positive aspects of the nursing profession were identified and the relevance of the nursing profession was worked out. The communication techniques were practiced in group exercises to facilitate the transfer into practice. In order to further consolidate the transfer into practice, the intervention group was given a “homework” exercise on positive patient communication for the training phase.

### Study Variables

#### Sense of Humor

To measure sense of humor, we used the *Sense of Humor Scale* [*SHS*, (17)] combined with the *Sense of Humor Scale parallel form* [*SHS-P* (30)] as recommended by Ruch and Heintz (30). Sense of humor showed a very good Cronbach's Alpha at both measuring times (t_0_ = 0.94 and t_1_ = 0.94). Sense of humor consists of the six subscales of *SHS* and *SHS-P* with a total of 48 items. Each of the six subscales contains a total of eight items. The subscales are *enjoyment of humor* (Cronbach's Alpha: t_0_ = 0.74 and t_1_ = 0.71; example item: “I enjoy funny sketches”), *laughter* (Cronbach's Alpha: t_0_ = 0.79 and t_1_ = 0.82; example item: “I feel comfortable laughing, even when others aren't”), *verbal humor* (Cronbach's Alpha: t_0_ = 0.85 and t_1_ = 0.85; example item: “I often make funny comments”), *finding humor in everyday life* (Cronbach's Alpha: t_0_ = 0.87 and t_1_ = 0.85; example item: “I can get something funny out of a lot of activities”), *laughing at yourself* (Cronbach's Alpha: t_0_ = 0.83 and t_1_ = 0.85; example item: “I find it easy to laugh when I am the butt of the joke”) and *humor under stress* (Cronbach's Alpha: t_0_ = 0.89 and t_1_ = 0.90; example item: “My sense of humor is for me a good way to cope with stress”). The items were measured on a 7-point-Likert scale from (1) “strong disapproval” to (7) “strong agreement.”

#### Perceived Stress

Perceived stress was measured with the *Perceived Stress Scale Questionnaire (PSQ)* by Fliege et al. (101). The *PSQ* (Cronbach's Alpha: t_1_ = 0.85) consists of a total of 20 items, which are divided into four subscales. The subscales of the *PSQ* are *tension, joy* (with inverted items), *worries* and *demands*. An example item of the *PSQ* is “You feel under pressure from deadlines.” The items were measured on a 4-point-Likert scale from (1) “almost never,” (2) “sometimes,” (3) “frequently” to (4) “most often.” Participants were instructed to relate their answers to their last 4 weeks at work.

#### Work Enjoyment

We assessed *work enjoyment* with the items of the subscale “*joy*” of the *PSQ* by Fliege et al. (101). High values represented more work enjoyment during practical training. The Cronbach's Alpha of *work enjoyment during practical training* was t_1_= 0.65. An example item is “You have fun.”

#### Frequency of Flow Experience

The *frequency of flow* was assessed with the Flow Frequency Scale Bartzik and Peifer^1^ which contains 11 items on a 6-point-Likert scale with (1) “never,” (2) “almost never,” (3) “sometimes,” (4) “often,” (5) “very often,” (6) “(almost) always.” The instructions of the Flow Frequency items were: “Below you will find a number of questions about your daily work experience. Please rate how often or rarely you have had the experience in the last 2 weeks.” An example item reads: “How often have you experienced in the last 2 weeks at work that you were surprised how quickly time passed.” The Cronbach's Alpha (t_1_) can be described as very good with α = 0.87. The scale can be found in Supplementary Table 1.

#### Perceived Meaningfulness of Work

Perceived meaningfulness of work was measured with seven self-generated items, which are measured on a 6-point-Likert scale from (1) “do not agree” to (6) “fully agree” An example item of the perceived importance of the work is “My work is meaningful.” Cronbach's Alpha (t_1_) was very good with α = 0.85. The scale can be found in Supplementary Table 2.

#### Positive and Negative Affect

Positive and negative affect were assessed with the *Scale of Positive and Negative Experience (SPANE)* by Diener et al. (102) with a total of 12 items. For the humor intervention we adapted the instructions into “Please mark with a cross how you feel now, at this moment, according to the terms listed below.” Positive affect [*SPANE-P*; Cronbach's Alpha: t_(i0)_ = 0.87 and t_(i1)_ = 0.93; example item: “positive”] and negative affect [*SPANE-N*; Cronbach's Alpha: t_(i0)_ = 0.88 and t_(i1)_ = 0.82; example item: “sad”] were measured with six items each on a 5-point-Likert scale from (1) “not at all,” (3) “neutral” to (5) “very”.

#### Evaluation of the Humor Intervention

The reactions, learning experiences and attitudes regarding the humor intervention were measured with the Training Evaluation Inventory (TEI) by Ritzmann et al. (103) with 17 items. To evaluate the humor intervention, we used the scales for training outcome dimensions (subjective enjoyment [Cronbach's Alpha: t_(i1)_ = 0.85], perceived usefulness [Cronbach's Alpha: t_(i1)_ = 0.85], perceived difficulty [Cronbach's Alpha: t_(i1)_ = 0.83], subjective knowledge gain [Cronbach's Alpha: t_(i1)_ = 0.86], and attitude toward training [Cronbach's Alpha: t_(i1)_ = 0.72]). The subscales subjective enjoyment (example item: “Learning was fun”), perceived usefulness (example item: “Investing time in this intervention was useful”) and perceived difficulty (example item: “The contents were understandable”) represent level 1 (reactions) and level 2 (learning and attitude) are described with the subscales subjective knowledge gain (example item: “I will be able to remember the new topics well”) and attitude toward training (example item: “I will apply what I have learned in my daily work”). The items were assessed on a 5-point-Likert scale from (1) “does not apply at all” to (5) “fully applies.”

### Data Analysis

The data was analyzed using IBM SPSS statistics 26. To test the effectiveness of the intervention over time, we performed a repeated measures ANOVA in which we compared the intervention and control group with respect to their changes in their sense of humor from t_0_ to t_1_. The same approach was used with the subscales *enjoyment of humor, laughter, verbal humor, finding humor in everyday life, laughing at yourself* , and *humor under stress*. To assess the effect of the intervention on positive and negative affect, we used a paired *t*-test and report the effect size *d*_z_ (difference of the mean value of both measuring times divided by the standard deviation). In all analyses, we defined a significance level of *p* ≤ 0.050 to report statistically significant results. The mediation hypotheses were tested with the macro PROCESS by Hayes (104). All variables in the mediation models were z-standardized. According to Preacher and Hayes (105), the indirect effect *ab* was estimated to evaluate whether the humor intervention had an indirect effect *via* the sense of humor (t_1_) on the hypothesized outcome variables (perceived stress, frequency of flow, and perceived meaningfulness of work). We report a 95% confidence interval (n_bootstrap_ = 5,000) for the indirect effect.

## Results

### Descriptive Data and Intercorrelations

The descriptive data of the study variables divided into overall, intervention and control group for the measurement times t_0_ and t_1_ are presented in Table 2, and the intercorrelations can be taken from Table 3.

**Table 2:** Shows means, standard deviations of all study variables.

	**Variable**	***M* (*N*)**	***SD***	***M*_**CG**_ (*N_**CG**_*)**	***SD*_**CG**_**	***M*_**IG**_ (*N*_**IG**_)**	***SD*_**IG**_**
		**Overall sample**		**Control group (CG)**		**Intervention group (IG)**	
1	Sense of humor (t_0_)	4.91 (104)	0.782	4.88 (33)	0.536	4.92 (71)	0.876
2	Sense of humor (t_1_)	4.73 (94)	0.789	4.50 (31)	0.635	4.84 (63)	0.837
3	Enjoyment of humor (t_0_)	4.39 (104)	0.972	4.44 (33)	0.879	4.37 (71)	1.018
4	Enjoyment of humor (t_1_)	4.18 (94)	0.900	4.02 (31)	0.853	4.26 (63)	0.915
5	Finding humor in everyday life (t_0_)	5.19 (104)	0.963	5.11 (33)	0.645	5.22 (71)	1.082
6	Finding humor in everyday life (t_1_)	4.97 (94)	0.938	4.70 (31)	0.737	5.10 (63)	1.002
7	Laughing at yourself (t_0_)	5.41 (104)	0.992	5.28 (33)	0.806	5.47 (71)	1.068
8	Laughing at yourself (t_1_)	5.18 (94)	0.985	4.82 (31)	0.933	5.35 (63)	0.970
9	Laughter (t_0_)	5.25 (104)	0.934	5.17 (33)	0.792	5.29 (71)	0.996
10	Laughter (t_1_)	4.93 (94)	0.977	4.85 (31)	0.941	4.98 (63)	0.999
11	Verbal humor (t_0_)	4.42 (104)	1.215	4.59 (33)	0.875	4.37 (71)	1.342
12	Verbal humor (t_1_)	4.41 (94)	1.107	4.25 (31)	0.731	4.49 (63)	1.250
13	Humor under stress (t_0_)	4.78 (104)	1.122	4.68 (33)	0.956	4.83 (71)	1.195
14	Humor under stress (t_1_)	4.68 (94)	1.074	4.35 (31)	0.880	4.84 (63)	1.127
15	Perceived stress (t_1_)	2.27 (94)	0.410	2.30 (31)	0.358	2.26 (63)	0.436
16	Work enjoyment during practical training (t_1_)	2.63 (94)	0.534	2.49 (31)	0.412	2.70 (63)	0.576
17	Flow frequency (t_1_)	3.95 (93)	0.705	3.83 (30)	0.673	4.01 (63)	0.717
18	Perceived meaningfulness of work (t_1_)	4.82 (94)	0.829	4.68 (31)	0.812	4.88 (63)	0.835

*Scale-Range (1–7) = Sense of humor, Enjoyment of humor, Finding humor in everyday life, Laughing at yourself, Laughter, Verbal humor, Humor under stress; (1–4) = Perceived Stress, Work enjoyment during practical; (1–6) = Flow frequency, Perceived Meaningfulness of work; CG, control group; IG, intervention group; Measuring points: t_0_ = Baseline; t_1_ = 6 months after the humor intervention*.

**Table 3:** Intercorrelation of all study variables.

**Variable**		**1**		**2**		**3**		**4**		**5**		**6**		**7**		**8**		**9**		**10**		**11**		**12**		**13**		**14**		**15**		**16**		**17**		**18**
1	Sense of humor (t_0_)	1																																		
2	Sense of humor (t_1_)	0.74	^**^	1																																
3	Enjoyment of humor (t_0_)	0.47	^**^	0.30	^**^	1																														
4	Enjoyment of humor (t_1_)	0.42	^**^	0.54	^**^	0.66	^**^	1																												
5	Laughter (t_0_)	0.74	^**^	0.61	^**^	0.33	^**^	0.39	^**^	1																										
6	Laughter (t_1_)	0.57	^**^	0.82	^**^	0.19		0.43	^**^	0.77	^**^	1																								
7	Verbal humor (t_0_)	0.83	^**^	0.64	^**^	0.25	^*^	0.23	^*^	0.51	^**^	0.41	^**^	1																						
8	Verbal humor (t_1_)	0.64	^**^	0.84	^**^	0.20		0.32	^**^	0.46	^**^	0.63	^**^	0.72	^**^	1																				
9	Finding humor in everyday life (t_0_)	0.90	^**^	0.65	^**^	0.19		0.22		0.57	^**^	0.45	^**^	0.80	^**^	0.57	^**^	1																		
10	Finding humor in everyday life (t_1_)	0.72	^**^	0.89	^**^	0.16		0.30	^**^	0.57	^**^	0.66	^**^	0.67	^**^	0.74	^**^	0.74	^**^	1																
11	Laughing at yourself (t_0_)	0.79	^**^	0.65	^**^	0.11		0.24	^*^	0.55	^**^	0.51	^**^	0.63	^**^	0.54	^**^	0.77	^**^	0.65	^**^	1														
12	Laughing at yourself (t_1_)	0.58	^**^	0.82	^**^	0.10		0.29	^**^	0.35	^**^	0.60	^**^	0.53	^**^	0.62	^**^	0.57	^**^	0.76	^**^	0.72	^**^	1												
13	Humor under stress (t_0_)	0.80	^**^	0.51	^**^	0.28	^**^	0.23	^*^	0.44	^**^	0.32	^**^	0.57	^**^	0.41	^**^	0.76	^**^	0.50	^**^	0.54	^**^	0.33	^**^	1										
14	Humor under stress (t_1_)	0.56	^**^	0.81	^**^	0.16		0.28	^**^	0.37	^**^	0.55	^**^	0.43	^**^	0.62	^**^	0.54	^**^	0.75	^**^	0.43	^**^	0.61	^**^	0.60	^**^	1								
15	Perceived stress (t_1_)	−0.12		−0.22	^*^	0.02		−0.02		−0.12		−0.23	^*^	−0.07		−0.09		−0.13		−0.18		−0.19		−0.33	^**^	−0.08		−0.21	^*^	1						
16	Work enjoyment during practical training (t_1_)	0.21		0.38	^**^	0.13		0.19		0.28	^*^	0.36	^**^	0.05		0.26	^*^	0.16		0.33	^**^	0.10		0.31	^**^	0.24	^*^	0.37	^**^	−0.56	^**^	1				
17	Flow frequency (t_1_)	0.18		0.42	^**^	−0.01		0.08		0.22		0.35	^**^	0.11		0.27	^**^	0.19		0.39	^**^	0.08		0.37	^**^	0.23		0.50	^**^	−0.26	^*^	0.54	^**^	1		
18	Perceived meaningfulness of work (t_1_)	0.36	^*^	0.41	^**^	0.08		0.03		0.27	^*^	0.28	^**^	0.27	^*^	0.31	^**^	0.35	^**^	0.46	^**^	0.36	^**^	0.40	^**^	0.30	^**^	0.45	^**^	−0.37	^**^	0.58	^**^	0.48	^**^	1

** *p < 0.001*;

* *p < 0.050; Measuring points: t_0_ = Baseline; t_1_ = 6 months after the humor intervention*.

### Reactions, Subjective Learning Gain, and Attitude Toward the Humor Intervention

The humor intervention for the intervention group (*N*_IG_ = 70) shows descriptive values from *M* = 4.00 to *M* = 4.63 for the different training outcome dimensions, which shows an overall very positive assessment of our intervention by the participants. An overview of the descriptive values of the training outcome dimensions of Level 1 (reactions) and Level 2 (learning and attitudes) is shown in Table 4.

**Table 4:** Shows means and standard deviations of the training outcome dimensions.

**Variable**	***M***	***SD***
**Training outcome dimensions**		
**Level 1 (reactions)**		
1 Subjective enjoyment	4.25	0.671
2 Perceived usefulness	4.27	0.675
3 Perceived difficulty	4.63	0.467
**Level 2 (learning and attitude)**		
4 Subjective knowledge gain	4.00	0.758
5 Attitude toward training	4.26	0.642

*Measured with the TEI (103) on a 5-point Likert scale directly after the humor intervention [t_(i1)_]*.

#### Affect Before and After the Humor Intervention

**Figure 2:**
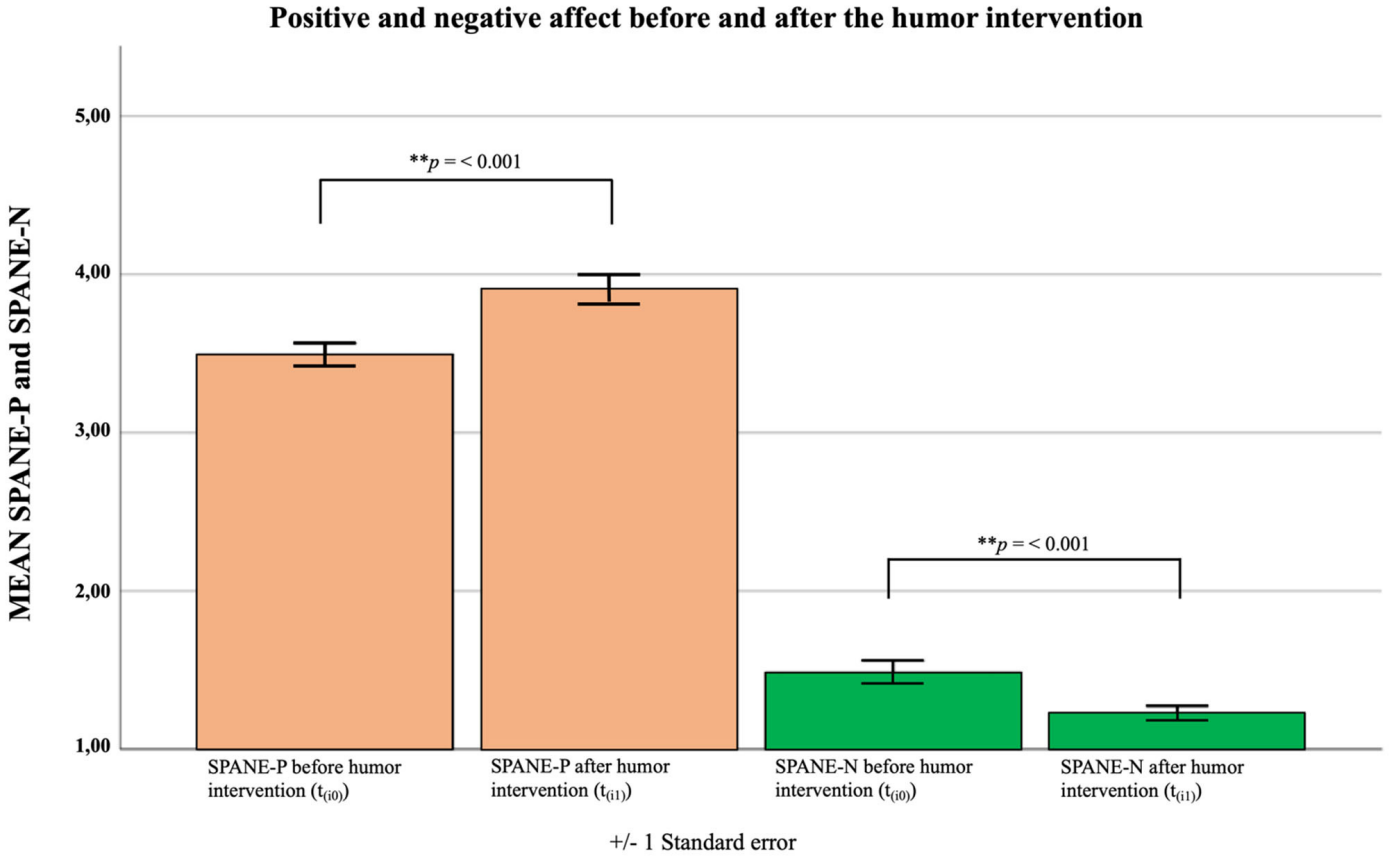
Affect before and after the humor intervention in the intervention group; SPANE-P, positive affect; SPANE-N, negative affect; Measuring points: t_(i0)_ = directly before the humor intervention; t_(i1)_ = directly after the humor intervention.

Positive affect [*M*_t(i0)_ = 3.49, *SD*_t(i0)_ = 0.588; *M*_t(i1)_ = 3.91, *SD*_t(i1)_ = 0.746] was significantly increased after the humor intervention [*t*_(66)_ = 5.81, *p* ≤ 0.001, *d*_z_ = 0.71], while negative affect was significantly decreased [*M*_t(i0)_ = 1.49, *SD*_t(i0)_ = 0.608; *M*_t(i1)_ = 1.23, *SD*_t(i1)_ = 0.374] after humor intervention [*t*_(66)_ = −4.28, *p* ≤ 0.001, *d*_z_ = −0.52]. The humor intervention resulted in an increase in positive affect and a decrease in negative affect (see Figure 2). The intercorrelations of acute changes in positive affect and negative affect due the humor intervention with sense of humor and its subscales, as well as perceived stress, work enjoyment, frequency of flow experience, and perceived meaningfulness of work as measured at t_1_, are depicted in Table 5.

**Table 5:** Intercorrelation of difference scores of affect at t_*i*_ with work experience 6 months after the humor intervention.

**Variable**		**1**		**2**	**3**		**4**		**5**		**6**		**7**		**8**		**9**		**10**		**11**		**12**		**13**
1	Difference score of positive affect (t_i1_-t_i0_)	1																							
2	Difference score of negative affect (t_i1_-t_i0_)	−0.36	^**^	1																					
3	Sense of humor (t_1_)	0.11		−0.03	1																				
4	Enjoyment of humor (t_1_)	0.29	^*^	−0.04	0.52	^**^	1																		
5	Laughter (t_1_)	0.10		−0.05	0.84	^**^	0.41	^**^	1																
6	Verbal humor (t_1_)	−0.00		−0.09	0.86	^**^	0.32	^*^	0.63	^**^	1														
7	Finding humor in everyday life (t_1_)	0.05		−0.05	0.92	^**^	0.31	^*^	0.73	^**^	0.80	^**^	1												
8	Laughing at yourself (t_1_)	0.00		−0.00	0.81	^**^	0.33	^**^	0.62	^**^	0.64	^**^	0.75	^**^	1										
9	Humor under stress (t_1_)	0.12		0.08	0.82	^**^	0.23		0.62	^**^	0.66	^**^	0.76	^**^	0.56	^**^	1								
10	Perceived stress (t_1_)	−0.05		0.04	−0.21		−0.12		−0.25		−0.07		−0.11		0.33	^**^	−0.18		1						
11	Work enjoyment during practical training (t_1_)	0.36	^*^	0.07	0.35	^**^	0.24		0.32	^**^	0.22		0.28	^*^	0.26	^*^	0.36	^**^	−0.53	^**^	1				
12	Flow frequency (t_1_)	0.23		−0.06	0.35	^**^	−0.02		0.27	^*^	0.26	^*^	0.33	^**^	0.27	^*^	0.51	^**^	−0.27	^*^	0.56	^**^	1		
13	Perceived meaningfulness of work (t_1_)	0.22		−0.02	0.41	^**^	0.06		0.27	^*^	0.37	^**^	0.43	^**^	0.36	^**^	0.45	^**^	−0.33	^**^	0.58	^**^	0.43	^**^	1

** *p < 0.001*;

* *p < 0.050; Measuring points: t_i1_-t_i0_ = Difference scores: measures are taken immediately before and immediately after the humor intervention; t_1_ = 6 months after the humor intervention*.

### Testing the Effectiveness of the Humor Intervention

First of all, there are significant effects of time in the overall group, showing that the sense of humor decreases from t_0_ to t_1_: Those significant main effects of time were found for *sense of humor* [*F*_(1,73)_ = 8.51, *p* = 0.005, η^2^ = 0.104], as well as for the subscales *finding humor in everyday life* [*F*_(1,73)_ = 7.69, *p* = 0.007, η^2^ = 0.095], *laughter* [*F*_(1,73)_ = 8.39, *p* = 0.005, η^2^ = 0.103], and *enjoyment of humor* [*F*_(1,73)_ = 8.22, *p* = 0.005, η^2^ = 0.101]. There are no significant main effects over time for *humor under stress* [*F*_(1,73)_ = 1.29, *p* = 0.260, η^2^ = 0.017], *verbal humor* [*F*_(1,73)_ = 2.28, *p* = 0.135, η^2^ = 0.030], and *laughing at yourself* [*F*_(1,73)_ = 3.46, *p* = 0.067, η^2^ = 0.045]. The group had no significant main effects.

*Testing Hypothesis 1: The humor intervention has a positive effect on the nurses' sense of humor and the six sense of humor habits*.

**Figure 3:**
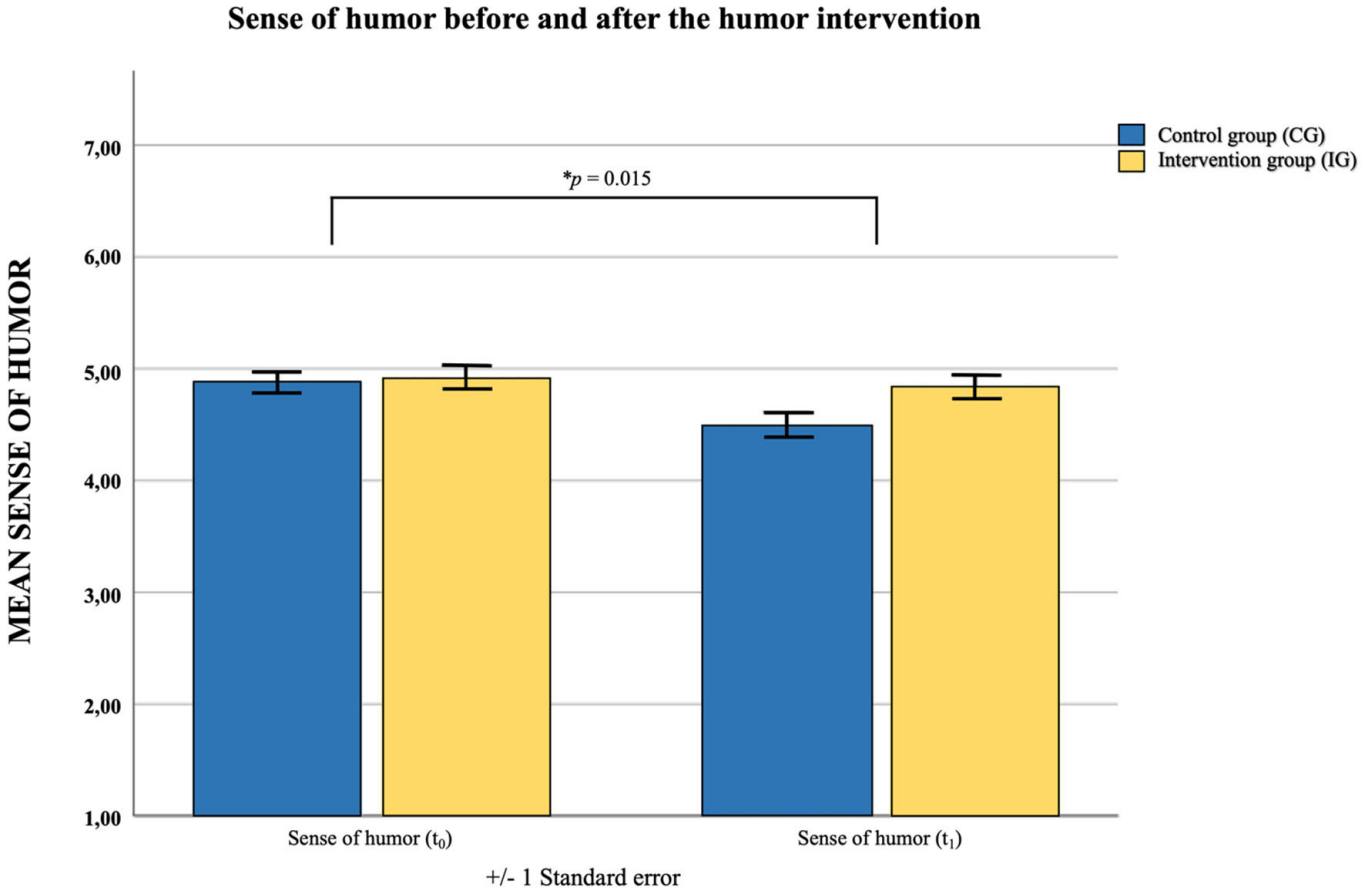
Sense of humor before and after the humor intervention comparing control group (CG) and intervention group (IG); Measuring points: t_0_ = Baseline; t_1_ = 6 months after the humor intervention; the interaction effect (*p* = 0.015) indicated that sense of humor decreased from t_0_ to t_1_ in the control group, but remained stable in the intervention group.

To test hypothesis 1, we looked at the interaction effects of time^*^group on the sense of humor variables from t_0_ to t_1_. Significant interaction effects were found on *sense of humor* [*F*_(1,73)_ = 6.26, *p* = 0.015, η^2^ = 0.079; see Figure 3], as well as on the subscales *finding humor in everyday life* [*F*_(1,73)_ = 5.29, *p* = 0.024, η^2^ = 0.068] and *verbal humor* [*F*_(1,73)_ = 10.94, *p* = 0.001, η^2^ = 0.130]. On these (sub)scales it was shown that sense of humor decreased from t_0_ to t_1_ in the control group, while it remained stable over time in the intervention group.

For *humor under stress* [*F*_(1,73)_ = 2.20, *p* = 0.142, η^2^ = 0.029], *laughing at yourself* [*F*_(1,73)_ = 2.34, *p* = 0.130, η^2^ = 0.031], *laughter* [*F*_(1,73)_ = 0.04, *p* = 0.842, η^2^ = 0.001], and *enjoyment of humor* [*F*_(1,73)_ = 2.80, *p* = 0.099, η^2^ = 0.037] no interaction effects with group and time could be found.

*Testing Hypothesis 2: Sense of humor mediates the effect of the humor intervention on perceived stress*.

**Figure 4:**
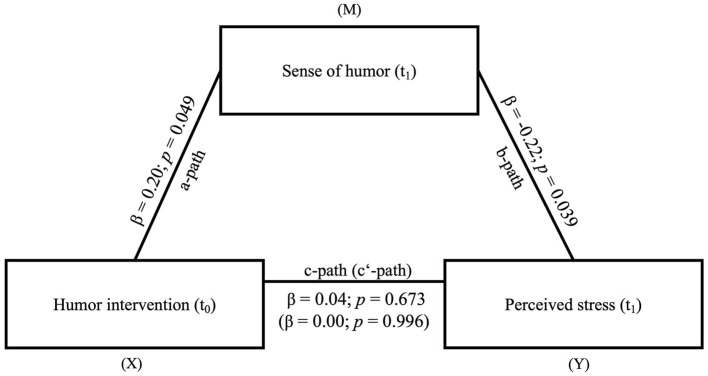
Mediation model of the effect of the humor intervention (X) on perceived stress (Y) *via* sense of humor (M). *N* = 94. The indirect effect from (X = independent variable) to (Y = dependent variable) *via* (M = mediator) was significant (β = −0.04, *SE* = 0.03, −0.11 < *CI* < 0.00); Measuring points: t_0_ = Baseline; t_1_ = 6 months after the humor intervention.

In the mediation model of hypothesis 2, the humor intervention (t_0_) is the independent variable, sense of humor (t_1_) is the mediator and perceived stress (t_1_) the dependent variable. The *a*-path (β = 0.20, *SE* = 0.10, *t* = 2.00, *p* = 0.049) and *b*-path (β = −0.22, *SE* = 0.10, *t* = −2.10, *p* = 0.039) were both significant. However, neither the total effect (β = −0.04, *SE* = 0.10, *t* = −0.42, *p* = 0.673), nor the direct effect (β = 0.00, *SE* = 0.10, *t* = 0.01, *p* = 0.996) or the indirect effect (β = −0.04, *SE* = 0.03, −0.11 < CI < 0.00) were significant. Accordingly, hypothesis 2 could not be confirmed (see Figure 4).

*Testing Hypothesis 3: Sense of humor mediates the effect of the humor intervention on work enjoyment during practical training*.

**Figure 5:**
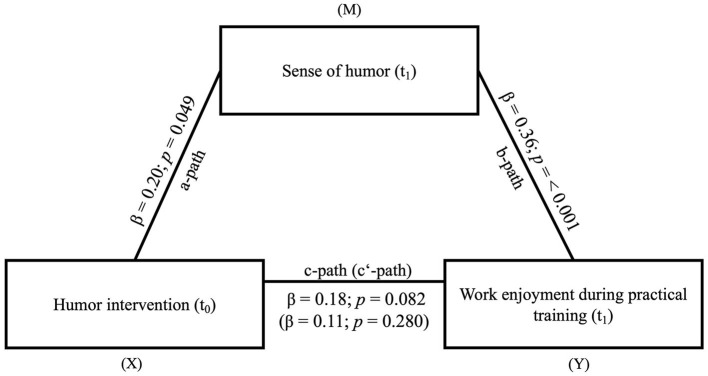
Mediation model of the effect of the humor intervention (X) on work enjoyment during practical training (Y) *via* sense of humor (M). *N* = 94. The indirect effect from (X = independent variable) to (Y = dependent variable) *via* (M = mediator) was significant (β = 0.07, *SE* = 0.04, 0.01 < *CI* < 0.15); Measuring points: t_0_ = Baseline; t_1_ = 6 months after the humor intervention.

To test hypothesis 3, the mediation model includes the humor intervention (t_0_) as independent variable, the sense of humor (t_1_) as mediator, and work enjoyment (t_1_) as dependent variable. We could show significant results for the *a*-path (β = 0.20, *SE* = 0.10, *t* = 2.00, *p* = 0.049) and *b*-path (β = 0.36, *SE* = 0.10, *t* = 3.68, *p* ≤ 0.001). The total effect (β = 0.18, *SE* = 0.10, *t* = 1.76, *p* = 0.082) and direct effect (β = 0.11, *SE* = 0.10, *t* = 1.09, *p* = 0.280) were not significant. However, we could show a significant indirect effect (β = 0.07, *SE* = 0.04, 0.01 < CI < 0.15). Sense of humor thus mediates a positive effect of the humor intervention on work enjoyment (see Figure 5).

*Testing Hypothesis 4: Sense of humor mediates the effect of the humor intervention on the frequency of flow experience*.

**Figure 6:**
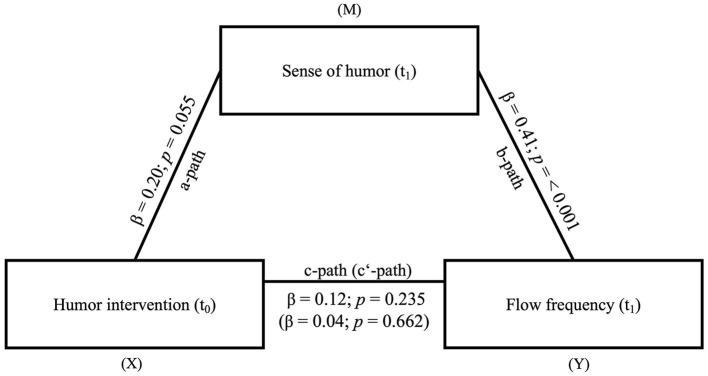
Mediation model of the effect of the humor intervention (X) on flow frequency (Y) via sense of humor (M). *N* = 93. The indirect effect from (X = independent variable) to (Y = dependent variable) *via* (M = mediator) was significant (β = 0.08, *SE* = 0.04, 0.01 < *CI* < 0.17); Measuring points: t_0_ = Baseline; t_1_ = 6 months after the humor intervention.

In the mediation model the humor intervention (t_0_) is the independent variable, sense of humor (t_1_) is the mediator and the frequency of flow (t_1_) is the dependent variable. While the *a*-path (β = 0.20, *SE* = 0.10, *t* = 1.94, *p* = 0.055) was just barely not significant, the *b*-path (β = 0.41, *SE* = 0.09, *t* = 4.18, *p* ≤ 0.001) was significant. The total effect (β = 0.12, *SE* = 0.10, *t* = 1.20, *p* = 0.235) and the direct effect (β = 0.04, *SE* = 0.10, *t* = 0.44, *p* = 0.662) were not significant. However, we found a significant indirect effect (β = 0.08, *SE* = 0.04, 0.01 < CI < 0.17) in the mediation model. The sense of humor thus transmits a positive effect of the humor intervention on flow frequency. For an overview, see Figure 6.

*Testing Hypothesis 5: Sense of humor mediates the effect of the humor intervention on the perceived meaningfulness of work*.

**Figure 7:**
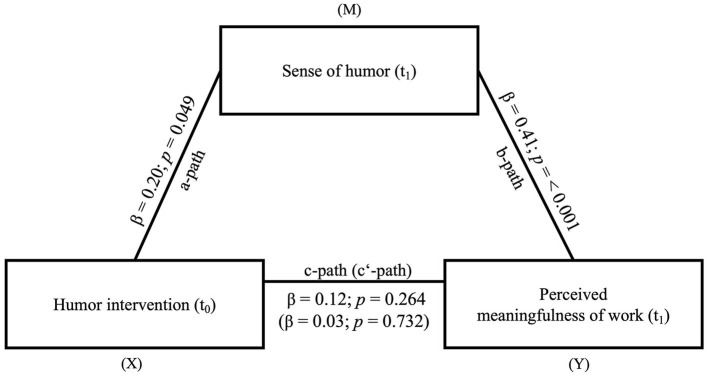
Mediation model of the effect of the humor intervention (X) on perceived meaningfulness of work (Y) via sense of humor (M). *N* = 94. The indirect effect from (X = independent variable) to (Y = dependent variable) *via* (M = mediator) was significant (β = 0.08, *SE* = 0.04, 0.01 < *CI* < 0.17); Measuring points: t_0_ = Baseline; t_1_ = 6 months after the humor intervention.

The mediation model involved sense of humor (t_1_) as mediator and the perceived meaningfulness of work (t_1_) as dependent variable. The independent variable in the mediation model is the humor intervention (t_1_). We could find significant results for the *a*-path (β = 0.20, *SE* = 0.10, *t* = 2.00, *p* = 0.049) and *b*-path (β = 0.41, *SE* = 0.10, *t* = 4.17, *p* ≤ 0.001). The total effect (β = 0.12, *SE* = 0.10, *t* = 1.12, *p* = 0.264) and the direct effect (β = 0.03, *SE* = 0.10, *t* = 0.34, *p* = 0.732) were not significant. However, we could show a significant indirect effect (β = 0.08, *SE* = 0.04, 0.01 < CI < 0.17). It can be concluded that sense of humor mediates a positive effect of the humor intervention on the perceived meaningfulness of work (see Figure 7). For an overview of the results of hypotheses 2–5, see Table 6.

**Table 6:** Mediation models of the effect of the humor intervention (X) via sense of humor (M) on the dependent variable (Y).

	***a*****-path**				***b*****-path**				***c*****-path**				***c'*****-path**				**Indirect effect**
**Variable (Y)**	**β**	***SE***	***t***		**β**	***SE***	***t***		**β**	***SE***	***t***		**β**	***SE***	***t***		**β*; CI***
Perceived stress (H2)	0.20	0.10	2.00	^*^	−0.22	0.10	−2.10	^*^	0.04	0.10	−0.42	^n.s.^	0.00	0.10	0.01	^n.s.^	β = −0.04; −0.11 < CI < 0.00
Work enjoyment during practical training (H3)	0.20	0.10	2.00	^*^	0.36	0.10	1.76	^**^	0.18	0.10	1.76	^n.s.^	0.11	0.10	1.09	^n.s.^	β = 0.07; 0.07 < CI < 0.15
Flow frequency (H4)	0.20	0.10	1.94	^n.s.^	0.41	0.09	4.18	^**^	0.12	0.10	1.20	^n.s.^	0.04	0.10	0.44	^n.s.^	β = 0.08; 0.01 < CI < 0.17
Perceived meaningfulness of work (H5)	0.20	0.10	2.00	^*^	0.41	0.10	4.17	^**^	0.12	0.10	1.12	^n.s.^	0.03	0.10	0.34	^n.s.^	β = 0.08; 0.01 < CI < 0.17

*(H), Hypothesis; n.s., not significant*;

** *p < 0.001*;

* *p < 0.050; X = independent variable (IG vs. CG); M = mediator (sense of humor); Y = dependent variable*.

## Discussion

### Summary of Results

In this study, we examined the effect of a humor intervention on the sense of humor in an intervention group with nurses in training, while a control group received no intervention. We were able to show in the results that the humor intervention had a protective effect on sense of humor in the intervention group, while the sense of humor in the control group decreased over a 6-month period. In addition, we found that the sense of humor mediated the effects of the humor intervention on work enjoyment, frequency of flow experience, and perceived meaningfulness of work. The sense of humor did not mediate the effect of the humor intervention on perceived stress. However, a direct negative effect of sense of humor on perceived stress was shown in the mediation model. Furthermore, we found that the humor intervention acutely increased positive affect and decreased negative affect. On a descriptive level of analysis, the nurses in training in the intervention group reported that they enjoyed the humor intervention, and the content of the humor intervention was also evaluated as useful for the nursing profession. Additionally, they rated the content of the humor intervention as easy to understand. The attitude toward the humor intervention was very positive and the humor intervention led to a subjective knowledge gain regarding its content. The nurses in training reported that their knowledge has expanded in the long term as a result of the humor intervention and that they are able to remember the content of the humor intervention well. It can be concluded that the implementation of the humor intervention in the context of nursing work was rated as very positive overall. Furthermore, we found positive correlations between the acute change in positive affect due to the intervention with enjoyment of humor and work enjoyment 6 months later.

### Discussion of the Hypotheses

In Hypothesis 1 we had postulated that the humor intervention would have a positive effect on the nurses' sense of humor and the six sense of humor habits. However, this was not exactly what we found: Instead of finding an increase of the sense of humor in the intervention group, we found it to be stable while it decreased in the control group. The finding of a decreased sense of humor in the group without intervention is, however, in line with other findings that, over the time of professional training, nurses show a decreased work satisfaction (106) and even the tendency to quit work (107), so such kinds of decreases are a typical although alarming phenomenon of the profession. Thus, we consider our finding that the humor intervention keeps the sense of humor stable during a 6-month post-measurement compared to a control group as a positive result that confirms Hypothesis 1. The finding is that sense of humor can be positively affected through training and is consistent with results from other studies (33, 54). We found the same result for some subscales of the sense of humor: “finding humor in everyday life” and “verbal humor.” This finding is highly plausible as the evaluated first module of our humor intervention addressed particularly positive communication in the nursing profession. The positive effect on the sense of humor habit “*verbal humor*” is promising here, as it improves people's communication skills and thus their ability to deal with conflicts (17). Its use will probably make it easier for nurses in training to establish contact with patients in the future. The sense of humor habit *finding humor in everyday life* can also help nurses in training to further develop the sense of humor in the future (17). For the subscale's *enjoyment of humor, laughing at yourself, laughter*, and *humor under stress*, we could not report any change due to our humor intervention. Later modules of the intervention will focus on other aspects of the sense of humor; at present, their effects on the outcome variables remain to be tested.

Our results of hypothesis 2—that sense of humor mediates the effect of the humor intervention on perceived stress—was not confirmed, as the indirect effect was not significant. However, both the a-path and b-path of the mediation model were significant in the predicted direction, i.e., the humor intervention had a positive effect on the sense of humor at t_1_ (a-path) and the sense of humor had a negative effect on perceived stress (b-path). Possibly, the sample size and, thus, the power of our study were not large enough to detect an existing effect. Furthermore, the focus of the intervention was on positive communication and contact with the patient. One study shows that direct contact with patients can be a stressor for nurses, but other stress factors can also be identified in the nursing profession, such as emotional demands from patients, uncomfortable work environments, time pressure, or administrative responsibilities (108). Accordingly, a multitude of stressors might have influenced the perception of stress, and future interventions should also address other potential stressors. We must also point out that stress management will be dealt with at a later stage in our training series “*Care for joy*,” and we might be able to confirm hypothesis 3 at a later point in time. Still, the finding that sense of humor was negatively associated with perceived stress (b-path) is nevertheless consistent with previous studies on humor and stress [see e.g., (14–18)] and underlines the potential of sense of humor as a coping strategy.

The results in this study confirm Hypothesis 3, i.e., that our humor intervention has an indirect effect on work enjoyment *via* sense of humor. This result is consistent with the results that the use of humor in the workplace can lead to greater work enjoyment (74). Such an increase in work enjoyment is associated with positive consequences such as increased performance and reduced psychological stress (65).

Furthermore, our study provides additional results on the as-yet scarce research on the relationship between humor and flow experience. The postulated indirect effect of the humor intervention *via* sense of humor on the frequency of flow (Hypothesis 4) could be confirmed. The effect of sense of humor on the frequency of flow experience is consistent with the results of the studies by Plester and Hutchison (87) and Bakker and van Woerkom (92), which described fun as a predictor for achieving flow experience. In the study by van Oortmerssen et al. (88) a small correlation between flow and humor was found, but no further effects of humor and flow could be reported. Our results are particularly relevant for the work context, because well-being (77–81) and job satisfaction (109) are positively influenced by flow experience.

Hypothesis 5, which postulated that the sense of humor mediates the effect of the humor intervention on the perceived meaningfulness of work, was also confirmed. To our knowledge, there are no studies so far that have investigated the relationship between perceived meaningfulness of work and humor. This study thus gives a first empirical support of this association. This association is in line with humor as a character strength belonging to the category of transcendence strengths (26). Transcendence strengths in the character strength model are defined as strengths that create meaning. Humor may help to look at the positive sides of life, and humor may similarly help people to perceive the good sides of their own profession.

In sum, hypotheses 2–5 provided evidence that sense of humor positively affects workplace experience. As outlined in the introduction, humor is related to positive emotions, which act as a buffer toward stress (40). The association of humor with positive emotions is also reflected in the brain: for example an MRI study reported that humor causes activation in the mesolimbic dopaminergic reward system (110) and rewards can lead to positive experiences such as positive emotions and joy (111). Furthermore, humor was found to reduce the stress hormone cortisol (112). In line with this, individuals who use humor as a stress coping method are more likely to see stressful situations as a challenge rather than a threat (113). Through such a stress-buffering effect, which is even visible physiologically, humor can contribute to positive work experiences and to an increase in job satisfaction (114).

### Implications for Nursing

Our results provide first, but promising evidence that humor interventions can have a positive impact when included in the training curriculum for prospective nurses. Sense of humor in the nursing context has many positive effects, such as reduced stress, increased work enjoyment, frequency of flow, and perceived meaningfulness of work. Therefore, one could also expect positive effects of humor interventions, not only for nurses in training, but also for trained nurses and other health care professionals like physicians and therapists.

In future implementations of the humor interventions, a booster/refresher session after the humor intervention could be helpful to consolidate learnings for more pronounced results. Refreshers have been shown to significantly increase training effectiveness (115). A potential refreshing intervention could be implemented using an accompanying mobile app. Such a mobile app could be used to send brief exercises to the participants which could help to ensure transfer into practice. Also, this app could contain summaries from the humor intervention and a forum in which users can share and discuss their experiences.

In general, literature shows that humor leads to an increase in well-being (32–35), which, however, depends on different humor styles: for example aggressive humor and self-defeating humor can even lead to a decrease in well-being (34). Accordingly, it is even more important that nurses are trained on the topic of humor, so that the humor styles hindering for well-being in the work context can be consciously avoided. Misapplied humor, also called “the dark side of humor,” can also have a negative impact on relationships between colleagues at work (116). On the other hand, good forms of humor can contribute to positive relationship building among colleagues (49). Positive relationships at work are important resources, and it has been shown that colleague support can contribute to staying in a job rather than quitting (117).

It can be concluded that humor is a promising intervention in the context of health care.

### Limitations and Future Research

There are some limitations in this study that we would like to discuss. First of all, our study included one cohort of nurses in training from two nursing schools. While all nurses of the cohort were included in the study, our sample was still relatively small, which has implications for the power of statistical analyses and the probability of finding significant effects (118, 119). In order to detect relationships and differences of a still reasonable effect size (i.e., to reduce the probability of the type II error), we decided to not apply Bonferroni correction. This implies a higher risk that the null hypothesis is rejected although it is true. At the same time, findings regarding positive effects on work experience were very consistent for the different constructs, so we are optimistic that our findings will be replicable in larger samples. Furthermore, when comparing the intervention group and the control group, it is noticeable that the control group was smaller than the intervention group. Of course, equal sample sizes would have been desirable. Unfortunately, the cohort in the nursing school, which served as control condition, was smaller than the intervention cohort. Still, we consider the findings as first evidence for the effectiveness of a humor intervention for nurses in training. Future studies should add upon our initial results and aim at a larger sample size at best in a multicentric study to validate and generalize findings.

Another potential limitation of our study is that the possibility of randomization was limited. Students of one school were automatically assigned to the intervention group, students from the other school to the control group. This was necessary for several reasons: first, students are based in fixed classes, doing their training together. This means that from an organizational viewpoint, it would have been difficult to separate classmates. Second, even if classmates would have been separated, it is likely that students would have discussed their learnings with their classmates, which could have affected the results (crossover-effects). Therefore, we decided to separate students by school. Having baseline measures of both schools, we consider this a minor problem. Still, future investigations that apply a multicentric approach will be able to overcome this potential limitation.

Furthermore, we see potential for the improvement of our intervention: while the intervention group was given a “homework” exercise for the practice phase, we believe that a refresher session within the 6 months between the first and second measurement would increase the effects. This could also be done with a mobile app, reminding the participants of the contents of the intervention and providing small refresher tasks.

Finally, we want to address the statistical analyses conducted in this study: We have reported separate mediation models instead of one holistic model. This could be done using structural equation modeling. Again, a larger sample size would be necessary for such an endeavor.

Another potential future line of research is the link between humor and stress. There is as yet very little research in this area and the mechanisms of how humor reduces stress are not yet well-understood. One potential mechanism could be the concept of flow Bartzik and Peifer^2^. Flow was found to occur when a stress-relevant situation is re-interpreted as a pleasant challenge (120). Humor could act as a resource that helps to re-interpret an undesirable situation into a more favorable one, i.e., it could help reaching flow in stress-relevant situations (121)^1^. Future research should further explore this and other potential mechanisms explaining the link between humor and stress.

## Data Availability Statement

The raw anonymized data supporting the conclusions of this article will be made available by the authors upon request and in accordance with the ethical consent provided by participants.

## Ethics Statement

This study involved human participants and was reviewed and approved by the local ethics committee at the Faculty of Psychology, Ruhr University Bochum, Germany. Written informed consent to participate in this study was provided by the participants.

## Author Contributions

MBa, CP, AB, SH, AD-D, and PA carried out the study. MBa and CP developed the theory, wrote the methods and discussion. MBa performed the computations and wrote the results. CP supervised the concept and findings of this work. All authors conceived of the presented idea, discussed the results, and contributed to the final manuscript.

## Conflict of Interest

GK, PA, and AD-D were employed by the company Alexianer GmbH. The remaining authors declare that the research was conducted in the absence of any commercial or financial relationships that could be construed as a potential conflict of interest.

## Footnotes

^1^Bartzik M, Peifer C. Flow Frequency Scale (FFS) (in preparation).

^2^Bartzik M, Peifer C. On the relationships between humour, stress and flow experience – Introducing the Humour-Flow Model (in revision).

## References

[1] Bundesagentur für Arbeit. Fachkräfteengpassanalyse. In: Berichte: Blickpunkt Arbeitsmarkt. (2017). Available online at: https://statistik.arbeitsagentur.de/Statistikdaten/Detail/201712/arbeitsmarktberichte/fk-engpassanalyse/fk-engpassanalyse-d-0-201712-pdf.pdf

[2] Bundesagentur für Arbeit. Arbeitsmarktsituation im Pflegebereich. (2018). Available online at: http://statistik.arbeitsagentur.de

[3] Bundesagentur für Arbeit. Arbeitsmarktsituation im Pflegebereich. In: Berichte: Blickpunkt Arbeitsmarkt. (2020). Available online at: www.statistik.arbeitsagentur.de

[4] Hornung J. Nachhaltiges Personalmanagement in der Pflege. Das 5-Säulen Konzept. Berlin: Springer-Verlag (2013).

[5] Hasselhorn HM Müller BH. Arbeitsbelastung und -beanspruchung bei Pflegepersonal in Europa — Ergebnisse der Next-Studie. Fehlzeiten Rep. (2005) 2004:21–47. 10.1007/3-540-27051-5_2

[6] Hayes LJ O'Brien-Pallas L Duffield C Shamian J Buchan J Hughes F. Nurse turnover: a literature review - an update. Int J Nurs Stud. (2012) 49:887–905. 10.1016/j.ijnurstu.2011.10.001 22019402

[7] Chiang YM Chang Y. Stress, depression, and intention to leave among nurses in different medical units: implications for healthcare management/nursing practice. Health Policy. (2012) 108:149–57. 10.1016/j.healthpol.2012.08.027 23017221

[8] Choi JS Kim KM. Effects of nursing organizational culture and job stress on Korean infection control nurses' turnover intention. Am J Infection Control. (2020) 48:1404–6. 10.1016/j.ajic.2020.04.002 32289344 PMC7151524

[9] Schmitz N Neumann W Oppermann R. Stress, burnout and locus of control in German nurses. Int J Nurs Stud. (2000) 37:95–9. 10.1016/S0020-7489(99)00069-3 10684950

[10] Khamisa N Oldenburg B Peltzer K Ilic D. Work related stress, burnout, job satisfaction and general health of nurses. Int J Environ Res Public Health. (2015) 12:652–66. 10.3390/ijerph120100652 25588157 PMC4306884

[11] Vahey DC Aiken LH Sloane DM Clarke SP Vargas D. Nurse burnout and patient satisfaction. Med Care. (2004) 42(2 Suppl.):II57–66. 10.1097/01.mlr.0000109126.50398.5a 14734943 PMC2904602

[12] Estryn-Behar M Kaminski M Peigne E Bonnet N Vaichere E Gozlan C. Stress at work and mental health status among female hospital workers. Br J Indus Med. (1990) 47:20–8. 10.1136/oem.47.1.20 2310704 PMC1035090

[13] Roberts RK Grubb PL. The consequences of nursing stress and need for integrated solutions. Rehabil Nurs. (2014) 39:62–9. 10.1002/rnj.97 23696492 PMC4664060

[14] Bennett HJ. Humor in medicine. South Med J. (2003) 96:1257–61. 10.1097/01.SMJ.0000066657.70073.14 14696878

[15] Martin RA. Sense of humor and physical health: theoretical issues, recent findings, future directions. Humor Int J Humor Res. (2004) 17:1–19. 10.1515/humr.2004.005

[16] Martin RA Lefcourt HM. Sense of humor as a moderator of the relation between stressors and moods. J. Personal. Soc. Psychol. (1983) 45:1312–24. 10.1037/0022-3514.45.6.1313

[17] McGhee P. Humor as Survival Training for a Stressed-Out World: The 7 Humor Habits Program. Bloomington: AuthorHouse (2010).

[18] Putz D Breuer K. The stress-reducing effect of employee's and supervisor's positive humor at work. Wirtschaftspsychologie. (2017) 19:39–50.

[19] Ruch W Proyer RT Harzer C Park N Peterson C Seligman MEP. Values in action inventory of strengths (VIA-IS): adaptation and validation of the German version and the development of a peer-rating form. J Individual Differ. (2010) 31:138–49. 10.1027/1614-0001/a000022

[20] Scheel T. Definitions, theories, and measurement of humor. In: Scheel T Gockel C, editors. Humor at Work in Teams, Leadership, Negotiations, Learning and Health. Cham: Springer (2017). p. 9–29.

[21] Martin RA. The Psychology of Humor: An Integrative Approach. 1st ed. Burlington, MA: Elsevier Science (2007).

[22] Ruch W. Temperament, eysenck's pen system, humor-related traits. Humor. (1994) 7:209–44. 10.1515/humr.1994.7.3.209

[23] Ruch W. Measurement approaches to the sense of humor: introduction and overview. Humor. (1996) 9:239–50. 10.1515/humr.1996.9.3-4.239

[24] Ruch W. Psychology of humor. In: Raskin V, editor. The Primer of Humor Research. Berlin: Mouton de Gruyter (2008). p. 17–100.

[25] Martin RA. Sense of humor. In: Lopez SJ Snyder CR, editors. Positive Psychological Assessment: A Handbook of Models and Measures. Washington, DC: American Psychological Association (2003). p. 313–26.

[26] Peterson C Seligman MEP. Character Strengths and Virtues: A Handbook and Classification. New York, NY; Washington, DC: Oxford University Press; American Psychological Association (2004).

[27] Seligman MEP Steen TA Park N Peterson C. Positive psychology progress: empirical validation of interventions. Am Psychol. (2005) 60:410–21. 10.1037/0003-066X.60.5.410 16045394

[28] Müller L Ruch W. Humor and strengths of character. J Positive Psychol. (2011) 6:368–76. 10.1080/17439760.2011.592508

[29] McGhee P. Health, Healing and the Amuse System: Humor as Survival Training. Dubuque, IA: Kendall/Hunt (1996).

[30] Ruch W Heintz S. Psychometric evaluation of the revised sense of humor scale and the construction of a parallel form. Humor. (2018) 31:235–57. 10.1515/humor-2016-0085

[31] Meyer JC. Humor as a double-edged sword: four functions of humor in communication. Commun Theory. (2000) 10:310–31. 10.1111/j.1468-2885.2000.tb00194.x

[32] Cann A Collette C. Sense of humor, stable affect, psychological well-being. Europe J Psychol. (2014) 10:464–79. 10.5964/ejop.v10i3.746

[33] Crawford SA Caltabiano NJ. Promoting emotional well-being through the use of humour. J Positive Psychol. (2011) 6:237–52. 10.1080/17439760.2011.577087

[34] Jiang F Lu S Jiang T Jia H. Does the relation between humor styles and subjective well-being vary across culture and age? A meta-analysis. Front Psychol. (2020) 11:2213. 10.3389/fpsyg.2020.02213 33071846 PMC7536505

[35] Proyer RT Ruch W Müller L. Sinn für Humor bei Älteren: untersuchungen mit einer deutschen Fassung der Sense-of-Humor-Scale. Zeitschrift Fur Gerontologie Und Geriatrie. (2010) 43:19–24. 10.1007/s00391-009-0082-0 20012065

[36] Robert C Wilbanks JE. The wheel model of humor: humor events and affect in organizations. Human Relat. (2012) 65:1071–99. 10.1177/0018726711433133

[37] Szabo A. The acute effects of humor and exercise on mood and anxiety. J Leisure Res. (2003) 35:152–62. 10.1080/00222216.2003.11949988

[38] Wagner L Gander F Proyer RT Ruch W. Character strengths and PERMA: investigating the relationships of character strengths with a multidimensional framework of well-being. Appl Res Qual Life. (2020) 15:307–28. 10.1007/s11482-018-9695-z

[39] Seligman MEP. Flourish: A Visionary New Understanding of Happiness and Well-Being. New York, NY: Free Press (2011).

[40] McGhee P. Humor the Lighter Path to Resilience and Health. Bloomington: AuthorHouse (2010).

[41] McGhee P. Humor als copingstrategie. In: Wild B, editor. Humor in Psychiatrie und Psychotherapie: Neurobiologie - Methoden – Praxis. 2nd ed. Stuttgart: Schattauer (2016). p. 208–28.

[42] Li Z.-S. Hasson F. Resilience, stress, and psychological well-being in nursing students: a systematic review. Nurse Educ Today. (2020) 90:104440. 10.1016/j.nedt.2020.104440 32353643

[43] Scholl JC. The use of humor to promote patient-centered care. J Appl Commun Res. (2007) 35:156–76. 10.1080/00909880701262658

[44] Bauer M Geront M. The use of humor in addressing the sexuality of elderly nursing home residents. Sexuality Disability. (1999) 17:147–55. 10.1023/A:1021424401601

[45] Consoli AJ Blears K Bunge EL Mandil J Sharma H Whaling KM. Integrating culture, pedagogy, and humor in CBT with anxious and depressed youth. Prac Innov. (2018) 3:138–51. 10.1037/pri0000069

[46] Tanay MA Wiseman T Roberts J Ream E. A time to weep and a time to laugh: humour in the nurse-patient relationship in an adult cancer setting. Support Care Cancer. (2014) 22:1295–301. 10.1007/s00520-013-2084-0 24346848

[47] Greenberg M. Therapeutic play: developing humor in the nurse-patient relationship. J N Y State Nurses Assoc. (2003) 34:25–31. 14639778

[48] Sousa LMM Marques-Vieira CMA Antunes AV Frade M de FG Severino SPS. Humor intervention in the nurse-patient interaction. Rev Brasil Enfermagem. (2019) 72:1078–85. 10.1590/0034-7167-2018-0609 31432968

[49] Beck CT. Humor in nursing practice: a phenomenological study. Int J Nurs Stud. (1997) 34:346–52. 10.1016/S0020-7489(97)00026-6 9559383

[50] McCreaddie M Wiggins S. The purpose and function of humour in health, health care and nursing: a narrative review. J Adv Nurs. (2008) 61:584–95. 10.1111/j.1365-2648.2007.04548.x 18302600

[51] Frankenfield PK. The power of humor and play as nursing interventions for a child with cancer: a case report. J Pediatric Oncol Nurs. (1996) 13:15–20. 10.1016/S1043-4542(96)90085-5 8904462

[52] Tan T Schneider MA. Humor as a coping strategy for adult-child caregivers of individuals with alzheimer's disease. Geriatric Nurs. (2009) 30:397–408. 10.1016/j.gerinurse.2009.09.004 19963149

[53] Falkenberg I McGhee P Wild B. Humorfähigkeiten trainieren: Manual für die psychiatrisch-psychotherapeutische Praxis. Stuttgart: Schattauer (2013).

[54] Hofmann J Giuliani F. Forschung: humor ist trainierbar. In: Müller C, editor. HumorCare: Das Heiterkeitsbuch für Pflege- und Gesundheitsberufe. Bern: Hoegrefe Verlag (2019). p. 207–24.

[55] Ruch W Hofmann J Rusch S Stolz H. Training the sense of humor with the 7 Humor Habits Program and satisfaction with life. Humor. (2018) 31:287–309. 10.1515/humor-2017-0099

[56] Wellenzohn S Proyer RT Ruch W. Who benefits from humor-based positive psychology interventions? The moderating effects of personality traits and sense of humor. Front Psychol. (2018) 9:821. 10.3389/fpsyg.2018.00821 29892252 PMC5985328

[57] Barrows HS. Problem-based learning in medicine and beyond: a brief overview. New Directions Teach Learn. (1996) 1996:3–12. 10.1002/tl.37219966804

[58] Merrill MD. First principles of instruction. Educ Technol Res Dev. (2002) 50:43–59. 10.1007/BF02505024

[59] Dochy F Segers M Van den Bossche P Gijbels D. Effects of problem-based learning: a meta-analysis. Learn Instruct. (2003) 13:533–68. 10.1016/S0959-4752(02)00025-7

[60] Lazarus RS Folkman S. Stress, Appraisal, and Coping. Springer (1984). Available online at: http://www.dawsonera.com/depp/reader/protected/external/AbstractView/S9780826141927

[61] Warner SL. Humor: a coping response for student nurses. Arch Psychiatric Nurs. (1991) 5:10–6. 10.1016/0883-9417(91)90004-O 2039275

[62] Umucu E Lee B. Examining the impact of COVID-19 on stress and coping strategies in individuals with disabilities and chronic conditions. Rehabil Psychol. (2020) 65:193–8. 10.1037/rep0000328 32406739

[63] Canestrari C Bongelli R Fermani A Riccioni I Bertolazzi A Muzi M. Coronavirus disease stress among Italian healthcare workers: the role of coping humor. Front Psychol. (2021) 11:601574. 10.3389/fpsyg.2020.601574 33569023 PMC7868596

[64] Sun N Wei L Shi S Jiao D Song R Ma L. A qualitative study on the psychological experience of caregivers of COVID-19 patients. Am J Infection Control. (2020) 48:592–8. 10.1016/j.ajic.2020.03.018 32334904 PMC7141468

[65] Graves LM Ruderman MN Ohlott PJ Weber TJ. Driven to work and enjoyment of work: effects on managers' outcomes. J Manage. (2012) 38:1655–80. 10.1177/0149206310363612

[66] Wilkes L Doull M Ng Chok H Mashingaidze G. Enjoyment in nursing - experiences from the clinical milieu. J Clin Nurs. (2016) 25:656–63. 10.1111/jocn.12981 26526562

[67] Martínez-Martí ML Ruch W. Character strengths and well-being across the life span: data from a representative sample of German-speaking adults living in Switzerland. Front Psychol. (2014) 5:1–10. 10.3389/fpsyg.2014.01253 25408678 PMC4219388

[68] Bakker AB. The work-related flow inventory: construction and initial validation of the WOLF. J Vocational Behav. (2008) 72:400–14. 10.1016/j.jvb.2007.11.007

[69] Kafetsios K Zampetakis LA. Emotional intelligence and job satisfaction: testing the mediatory role of positive and negative affect at work. Personal Individual Differ. (2008) 44:712–22. 10.1016/j.paid.2007.10.004

[70] Moè A Pazzaglia F Ronconi L. When being able is not enough. The combined value of positive affect and self-efficacy for job satisfaction in teaching. Teaching Teacher Educ. (2010) 26:1145–53. 10.1016/j.tate.2010.02.010

[71] Cook JD Hepworth SJ Wall TD Warr PB. The Experience of Work: A Compendium and Review of 249 Measures and Their Use. San Diego, CA: Academic Press Limited (1981).

[72] Mesmer-Magnus J Glew DJ Viswesvaran C. A meta-analysis of positive humor in the workplace. J Manage Psychol. (2012) 27:155–90. 10.1108/02683941211199554

[73] Robert C Da Motta Veiga SP. Conversational humor and job satisfaction at work: exploring the role of humor production, appreciation, positive affect. Humor. (2017) 30:417–38. 10.1515/humor-2017-0034

[74] Ghaffari F Dehghan-Nayeri N Shali M. Nurses' experiences of humour in clinical settings. Int J Afr Nurs Sci. (2015) 29:182. 10.1016/j.ijans.2014.06.004 26034735 PMC4431435

[75] Csikszentmihalyi M. Beyond Boredom and Anxiety. San Francisco, CA: Jossey-Bass Publishers (1975).

[76] Peifer C Wolters G. Bei der Arbeit im Fluss sein: Konsequenzen und Voraussetzungen von Flow-Erleben am Arbeitsplatz. Wirtschaftspsychologie. (2017) 19:6–22.

[77] Asakawa K. Japanese college students: how do they. J Happiness Stud. (2004) 5:123–54. 10.1023/B:JOHS.0000035915.97836.89

[78] Asakawa K. Flow experience, culture, and well-being: how do autotelic Japanese college students feel, behave, and think in their daily lives? J Happiness Stud. (2010) 11:205–23. 10.1007/s10902-008-9132-3

[79] Bartzik M Wolters G Peifer C. Flow-Erleben im Arbeitskontext? – Eine Untersuchung der Zusammenhänge von affektivem tätigkeitsbezogenem Commitment mit Stress, Leistung und subjektivem Wohlbefinden. In: Brohm-Badry M Franz VS Peifer C, editors. Zusammen wachsen – Förderung der positiv-psychologischen Entwicklung von Individuum, Organisation und Gesellschaft. Nachwuchsforschung der DGPPF, Band II. Lengerich: Pabst Science Publishers (2020). p. 51–71.

[80] Bassi M Steca P Monzani D Greco A Delle Fave. A. Personality and optimal experience in adolescence: implications for well-being and development. J Happiness Stud. (2014) 15:829–43. 10.1007/s10902-013-9451-x

[81] Rivkin W Diestel S Schmidt KH. Which daily experiences can foster well-being at work? A diary study on the interplay between flow experiences, affective commitment, self-control demands. J Occup Health Psychol. (2018) 23:99–111. 10.1037/ocp0000039 27101337

[82] Christandl F Mierke K Peifer C. Time flows: manipulations of subjective time progression affect recalled flow and performance in a subsequent task. J Exp Soc Psychol. (2018) 74:246–56. 10.1016/j.jesp.2017.09.015

[83] Engeser S Rheinberg F. Flow, performance and moderators of challenge-skill balance. Motivation Emotion. (2008) 32:158–72. 10.1007/s11031-008-9102-4

[84] Peifer C Zipp G. All at once? The effects of multitasking behavior on flow and subjective performance. Euro J Work Org Psychol. (2019) 28:682–90. 10.1080/1359432X.2019.1647168

[85] Peifer C. Psychophysiological correlates of flow experience. In: Engeser S, editor. Advances in Flow Research. 1st ed. New York, NY: Springer (2012). p. 139–64.

[86] Plester B Cooper-Thomas H Winquist J. The fun paradox. Employee Relat. (2015) 37:380–98. 10.1108/ER-04-2013-0037

[87] Plester B Hutchison A. Fun times: the relationship between fun and workplace engagement. Employee Relat. (2016) 38:332–50. 10.1108/ER-03-2014-0027

[88] van Oortmerssen LA Caniëls MCJ van Assen MF. Coping with work stressors and paving the way for flow: challenge and hindrance demands, humor, and cynicism. J Happiness Stud. (2020) 21:2257–77. 10.1007/s10902-019-00177-9

[89] Collins AL Sarkisian N Winner E. Flow and happiness in later life: an investigation into the role of daily and weekly flow experiences. J Happiness Stud. (2009) 10:703–19. 10.1007/s10902-008-9116-3

[90] Eisenberger R Jones JR Stinglhamber F Shanock L Randall AT. Flow experiences at work: for high need achievers alone? J Org Behav. (2005) 26:755–75. 10.1002/job.337

[91] Fullagar CJ Kelloway EK. Flow at work: an experience sampling approach. J Occupat Org Psychol. (2009) 82:595–615. 10.1348/096317908X357903

[92] Bakker AB van Woerkom. M. Flow at work: a self-determination perspective. Occupat Health Sci. (2017) 1:47–65. 10.1007/s41542-017-0003-3

[93] Li D Browne GJ. The role of need for cognition and mood in online flow experience. J Computer Information Syst. (2006) 46:11–7.

[94] Hackman JR Oldham GR. Motivation through the design of work: test of a theory. Org Behav Human Perform. (1976) 16:250–79. 10.1016/0030-5073(76)90016-7

[95] Rosso BD Dekas KH Wrzesniewski A. On the meaning of work: a theoretical integration and review. Res Org Behav. (2010) 30:91–127. 10.1016/j.riob.2010.09.00130356783

[96] Geldenhuys M Łaba K Venter CM. Meaningful work, work engagement and organisational commitment. SA J Indus Psychol. (2014) 40:1–10. 10.4102/sajip.v40i1.1098

[97] Leufstadius C Eklund M Erlandsson LK. Meaningfulness in work - Experiences among employed individuals with persistent mental illness. Work. (2009) 34:21–32. 10.3233/WOR-2009-0899 19923673

[98] Arnold KA Turner N Barling J Kelloway EK McKee MC. Transformational leadership and psychological well-being: the mediating role of meaningful work. J Occupat Health Psychol. (2007) 12:193–203. 10.1037/1076-8998.12.3.193 17638487

[99] Steger MF Dik BJ Duffy RD. Measuring meaningful work: the work and meaning inventory (WAMI). J Career Assessment. (2012) 20:322–37. 10.1177/1069072711436160

[100] Bjarnadottir A. Work engagement among nurses in relationally demanding Jobs in the hospital sector. Nordic J Nurs Res. (2011) 31:30–4. 10.1177/010740831103100307

[101] Fliege H Rose M Arck P Levenstein S Klapp BF. Validierung des “Perceived Stress Questionnaire” (PSQ) an einer deutschen Stichprobe. Diagnostica. (2001) 47:142–52. 10.1026//0012-1924.47.3.142

[102] Diener E Wirtz D Tov W Kim-Prieto C Choi D won Oishi S. New measures of well-being: flourishing and positive and negative feelings. Soc Indicators Res. (2009) 39:247–66. 10.1007/978-90-481-2354-4_12

[103] Ritzmann S Hagemann V Kluge A. The training evaluation inventory (TEI) - evaluation of training design and measurement of training outcomes for predicting training success. Vocations Learn. (2014) 7:41–73. 10.1007/s12186-013-9106-4

[104] Hayes AF. Introduction to Mediation, Moderation, and Conditional Process Analysis. 2nd ed. New York, NY: The Guilford Press (2018).

[105] Preacher KJ Hayes AF. Asymptotic and resampling strategies for assessing and comparing indirect effects in multiple mediator models. Behav Res Methods. (2008) 40:879–91. 10.3758/BRM.40.3.879 18697684

[106] Ma C-C Samuels ME Alexander JW. Factors that influence nurses' job satisfaction. J Nurs Administr. (2003) 33:293–9. 10.1097/00005110-200305000-00005 12792284

[107] Cortese CG. Job satisfaction of Italian nurses: an exploratory study. J Nurs Manage. (2007) 15:303–12. 10.1111/j.1365-2834.2007.00694.x 17359430

[108] McGrath A Reid N Boore J. Occupational stress in nursing. Int J Nurs Stud. (2003) 40:555–65. 10.1016/S0020-7489(03)00058-0 12828980

[109] Maeran R Cangiano F. Flow experience and job characteristics: analyzing the role of flow in job satisfaction. Test Psychometr Methodol Appl Psycholo. (2013) 20:13–26. 10.4473/TPM20.1.2

[110] Mobbs D Greicius MD Abdel-Azim E Menon V Reiss AL. Humor modulates the Mesolimbic Reward Centers. Neuron. (2003) 40:1041–8. 10.1016/S0896-6273(03)00751-7 14659102

[111] Schultz W. Neuronal reward and decision signals: from theories to data. Physiol Rev. (2015) 95:853–951. 10.1152/physrev.00023.2014 26109341 PMC4491543

[112] Savage BM Lujan HL Thipparthi RR DiCarlo SE. Humor, laughter, learning, and health! A brief review. Adv Physiol Educ. (2017) 41:341–7. 10.1152/advan.00030.2017 28679569

[113] Martin RA Ford TE. The Psychology of Humor: An Integrative Approach. 2nd ed. London: Academic Press (2018).

[114] Khamisa N Peltzer K Ilic D Oldenburg B. Effect of personal and work stress on burnout, job satisfaction and general health of hospital nurses in South Africa. Health SA Gesondheid. (2017) 22:252–8. 10.1016/j.hsag.2016.10.001

[115] Kluge A Frank B. Counteracting skill decay: four refresher interventions and their effect on skill and knowledge retention in a simulated process control task. Ergonomics. (2014) 57:175–90. 10.1080/00140139.2013.869357 24382262

[116] Plester B. The Complexity of Workplace Humour. Cham: Springer International Publishing (2016). 10.1007/978-3-319-24669-7

[117] De Clercq D Azeem MU Haq IU Bouckenooghe D. The stress-reducing effect of coworker support on turnover intentions: moderation by political ineptness and despotic leadership. J Bus Res. (2020) 111:12–24. 10.1016/j.jbusres.2020.01.064

[118] Bühner M Ziegler M. Statistik für Psychologen und Sozialwissenschaftler. Munich: Pearson Deutschland GmbH (2009).

[119] Neyman J Pearson ES. The testing of statistical hypotheses in relation to probabilities a priori. Math Proc Cambridge Philos Soc. (1933) 29:492–510. 10.1017/S030500410001152X

[120] Csikszentmihalyi M. Flow: The Psychology of Optimal Experience. New York, NY: Harper & Row (1990).

[121] Peifer C Tan J. The psychophysiology of flow experience. In: Peifer C Engeser S, editors. Advances in Flow Research. 2nd ed. Cham: Springer International Publishing (2021). p. 191–230.

