# Supplementary material for: Care for Joy: Evaluation of a Humor Intervention and Its Effects on Stress, Flow Experience, Work Enjoyment, and Meaningfulness of Work
Source: Front Public Health. 2021 Jul 2;9:667821. doi: 10.3389/fpubh.2021.667821 (PMC8283127; doi:10.3389/fpubh.2021.667821)
Supplement: Supplementary file 1 [file Table_1.pdf]

Care for joy: Evaluation of a humor intervention and its effects on stress, flow experience, work enjoyment and meaningfulness of work

Supplementary material

Marek Bartzik[1], Andreas Bentrup[2], Susanne Hill[2], Maria Bley[2], Eckart von Hirschhausen[2], Gerrit Krause[3], Peter Ahaus[3], Angelika Dahl-Dichmann[4] & Corinna Peifer[1]

[1] University of Lübeck, Department of Psychology, Research Group Work and Health, Lübeck, Germany

[2] Stiftung Humor Hilft Heilen (Foundation Humour Helps Healing), Bonn, Germany

[3] Alexianer GmbH, Münster, Germany

[4] Alexianer GmbH, Berlin, Germany

Table S1

Flow Frequency Scale (Bartzik and Peifer, in preparation)

**Instruction**	
Below you will find a number of questions **about your daily work**. Please rate "how often" or "rarely" you have had the experience in the **last two weeks**.<br><br>**How often have you experienced in the last two weeks at work, that …**	
**English items**	**German items**
1. … you had clear goals in mind.	1. … Sie klare Ziele vor Augen hatten.
2. … you received the right amount of feedback.	2. … Sie das richtige Maß an Feedback erhielten.
3. … you were optimally challenged.	3. … Sie optimal gefordert wurden.
4. … you were completely focused on what you were doing.	4. … Sie vollständig auf Ihr Tun konzentriert waren.
5. … your actions were fluent and smooth.	5. … Ihr Tun flüssig und glatt verlief.
6. … one step automatically resulted in the next.	6. … ein Schritt ganz automatisch den nächsten ergab.
7. … you had everything under control.	7. … Sie alles unter Kontrolle hatten.
8. … you were surprised how quickly time passed.	8. … Sie überrascht waren, wie schnell die Zeit verging.
9. … you were completely absorbed in an activity.	9. … Sie ganz in einer Tätigkeit aufgingen.
10. … you became one with an activity.	10. … Sie mit einer Tätigkeit eins geworden sind.
11. … you "merged" with an activity.	11. … Sie mit einer Tätigkeit „verschmolzen" sind.
**Scale**	
(1) = "*Never*", (2) = "*Almost never*",<br>(3) = "*Sometimes*", (4) = "*Often*",<br>(5) = "*Very Often*", (6) = "*(Almost) always*"	(1) = „*Nie*", (2) = „Fast nie",<br>(3) = „Manchmal", (4) = „Häufig",<br>(5) = „Sehr häufig", (6) = „*(Fast) immer*"

Table S2

Perceived meaningfulness of work (self-created items)

**Instruction**	
Please mark how strongly you agree with the statements below.	
**English items**	**German items**
1. My work is meaningful.	1. Meine Arbeit ist sinnvoll.
2. The work I do is important for me.	2. Die Arbeit, die ich tue, ist wichtig für mich.
3. My work fulfils me.	3. Meine Arbeit erfüllt mich.
4. My work is meaningful to others.	4. Meine Arbeit ist bedeutsam für andere.
5. My work is meaningful to society.	5. Meine Arbeit ist bedeutsam für die Gesellschaft.
6. My work is appreciated by patients and relatives.	6. Meine Arbeit wird von den Patienten und Angehörigen wertgeschätzt.
7. My work is appreciated by society.	7. Meine Arbeit wird von der Gesellschaft wertgeschätzt.
**Scale**	
(1) =”*do not agree*” to (6) = “*fully agree*”	(1) = „*stimme nicht zu*“ bis (6) = „*stimme voll und ganz zu*“
